# Identification of miRNAs with potential roles in regulation of anther development and male-sterility in *7B*-*1* male-sterile tomato mutant

**DOI:** 10.1186/s12864-015-2077-0

**Published:** 2015-10-28

**Authors:** Vahid Omidvar, Irina Mohorianu, Tamas Dalmay, Martin Fellner

**Affiliations:** Laboratory of Growth Regulators, Centre of the Region Haná for Biotechnological and Agricultural Research, Palacký University and Institute of Experimental Botany AS CR, Šlechtitelů 11, CZ-78371 Olomouc, Czech Republic; School of Computing Sciences, University of East Anglia, Norwich, NR4 7TJ UK; School of Biological Sciences, University of East Anglia, Norwich, NR4 7TJ UK

**Keywords:** *7B*-*1* mutation, *Solanum lycopersicum*, Male-sterility, Abiotic stress, Another development, Meiosis

## Abstract

**Background:**

The *7B*-*1* tomato line (*Solanum lycopersicum* cv. Rutgers) is a photoperiod-sensitive male-sterile mutant, with potential application in hybrid seed production. Small RNAs (sRNAs) in tomato have been mainly characterized in fruit development and ripening, but none have been studied with respect to flower development and regulation of male-sterility. Using sRNA sequencing, we identified miRNAs that are potentially involved in anther development and regulation of male-sterility in *7B*-*1* mutant.

**Results:**

Two sRNA libraries from *7B*-*1* and wild type (WT) anthers were sequenced and thirty two families of known miRNAs and 23 new miRNAs were identified in both libraries. MiR390, miR166, miR159 were up-regulated and miR530, miR167, miR164, miR396, miR168, miR393, miR8006 and two new miRNAs, miR#W and miR#M were down-regulated in *7B*-*1* anthers. Ta-siRNAs were not differentially expressed and likely not associated with *7B*-*1* male-sterility. miRNA targets with potential roles in anther development were validated using 5′-RACE. QPCR analysis showed differential expression of miRNA/target pairs of interest in anthers and stem of *7B*-*1*, suggesting that they may regulate different biological processes in these tissues. Expression level of most miRNA/target pairs showed negative correlation, except for few. In situ hybridization showed predominant expression of miR159, *GAMYBL1*, *PMEI* and *cystatin* in tapetum, tetrads and microspores.

**Conclusion:**

Overall, we identified miRNAs with potential roles in anther development and regulation of male-sterility in *7B*-*1*. A number of new miRNAs were also identified from tomato for the first time. Our data could be used as a benchmark for future studies of the molecular mechanisms of male-sterility in other crops.

**Electronic supplementary material:**

The online version of this article (doi:10.1186/s12864-015-2077-0) contains supplementary material, which is available to authorized users.

## Background

The spontaneous *7B*-*1* mutant in tomato (*Solanum lycopersicum* cv. Rutgers) is a photoperiod-dependent male-sterile line, where in long days flowers are male-sterile with stamens that are shrunken and produce non-viable microspores [1]. In short days, flowers are fertile and produce normal stamens and viable pollen. A proteomic study suggested that microsporogenesis in *7B*-*1* breaks down prior to the meiosis in microspore mother cells (MMCs), which was associated with altered levels of several important proteins involved in tapetum degeneration and MMCs development [2]. Compared to the WT, *7B*-*1* has a higher tolerance to various abiotic stresses, specifically under blue light, is less sensitive to light-induced inhibition (i.e., de-etiolation) of hypocotyl growth, to blue light-induced stomata opening [3], and has an elevated level of endogenous ABA, but less GAs, IAA, and CKs [4–6]. Fellner and Sawhney, [5] demonstrated that there was a defect in blue light perception in *7B*-*1*, which affected hormonal sensitivity and their endogenous levels. This information adds on to the fact that the *7B*-*1* is a complex mutation with its primary effect still unknown. As a stress-tolerant male-sterile mutant, *7B*-*1* is a valuable germplasm for hybrid tomato breeding [7].

Recent studies have documented important regulatory functions of sRNAs in plant growth and development. There are two main types of sRNAs based on their biogenesis: small interfering RNAs (siRNAs), and microRNAs (miRNAs). siRNAs are processed from perfectly double-stranded RNA (dsRNA) and comprise different classes; those produced from dsRNA synthesized by RNA dependent RNA polymerase 6 (RDR6) (*trans*-acting or ta-siRNAs) [8, 9], by RDR2 (heterochromatin siRNAs) [10], or by overlapping antisense mRNAs (natural antisense siRNAs) [11]. While ta-siRNAs target mRNAs similarly to miRNAs, heterochromatin siRNAs cause DNA methylation and/or heterochromatin formation that lead to transcriptional gene silencing [12].

Ta-siRNAs play essential roles in regulating plant development, metabolism and responses to biotic and abiotic stresses [13]. Four families of *TAS* genes comprising eight loci have been identified in *Arabidopsis thaliana*, among which miR173 targets both *TAS1a*/*b*/*c* family and *TAS2* locus, miR390 targets *TAS3a*/*b*/*c* family, while miR828 triggers the production of *TAS4*-derived ta-siRNAs [14–16]. *TAS3a*-derived tasi*ARFs*, 5´D7(+) and 5´D8(+), target several *ARFs*, including *ARF2*, *3* and *4* [14, 17], which were proposed to act as suppressors in auxin signaling pathway [18]. Recently, a fifth *TAS* family (*TAS5*) was identified from tomato, which is triggered by miR482 [19].

Plant miRNAs are typically 21 nucleotides (nt) long, which are derived from single-stranded RNA transcripts that have the ability to fold into imperfect stem-loop secondary structures. These hairpins are processed by the ribonuclease III-like enzyme Dicer (DCL1) into miRNA/miRNA* duplexes. One of the strands of the miRNA/miRNA* duplex (usually mature miRNA) is incorporated into the RNA induced silencing complex (RISC), which guides the RISC to recognize target mRNA based on sequence complementarity. Mature miRNAs suppress the expression of their target genes by cleavage of the target mRNAs or translational repression [20]. Many plant miRNAs are conserved among species and have been implicated in processes, such as development, signal transduction, abiotic stress tolerance, and resistance to pathogens [21–23]. High-throughput sequencing was first used to identify tomato sRNAs, including miRNAs from young green fruits and more recently from several developmental stages of the fruits [24–27].

Recently, there is a growing interest towards understanding the role of miRNAs in regulation of male-sterility in plants. Even though there is no evidence that any miRNAs directly cause male-sterility in plants, it was suggested that miRNAs and their target genes play important roles in regulation of male-sterility. Wei et al., [28] identified several conserved and new miRNAs, which were differentially expressed during anther development in a male-sterile cotton mutant. These miRNAs targeted *HD*-*Zip III*-like, *ARF4*, *AP2 and ACC oxidase 3* genes, which are key genes involved in hormone signaling, cell patterning, and anti-oxidant metabolism.

Zhang et al., [29] reported that miR156, miR159, miR164, miR166, miR172 and miR319 were differentially expressed in sporogenous cell, MMCs and microspores between a male-sterile cotton and its maintainer line. The predicted targets for these miRNAs were involved in cotton growth and development, signal transduction and metabolism pathways. Jiang et al., [30] identified 15 miRNAs, which were differentially expressed in pollens of a male-sterile line of *Brassica campestris* and its wild type. There are increasing evidences showing that the function of miRNAs, including miR156, miR159, miR164, miR167, miR172 and miR319 is crucial during flower development and microsporogenesis [31–33]. MiR159 directs the cleavage of *GAMYB*-related transcripts, which are involved in regulation of anther development and microsporogensis [34, 35]. Overexpression of miR159 in *Arabidopsis thaliana* resulted in sterile anthers [36]. MiR167 targets *ARF6*/*8*, which regulate ovule and anther development [31]. In *Arabidopsis thaliana*, overexpression of miR167 led to male-sterility [37]. Mutation in a sRNA locus in rice which produced a 21-nt sRNA has led to environment-conditioned male-sterility [38].

sRNAs in tomato have been mainly studied in fruits development and ripening process, and no report has yet documented their involvement in regulation of male-sterility. The main goal of our study was to investigate whether sRNA biogenesis is affected by *7B*-*1* mutation, and if sRNAs, particularly miRNAs, are associated or involved in anther development and regulation of male-sterility in *7B*-*1*. Known and new miRNAs, ta-siRNAs and their targets were identified and their expressions were studied in *7B*-*1* and WT anthers. Targets of miRNAs with potential roles in anther development and microsporogenesis were validated using 5′-RACE and localization of miRNAs was further analyzed by in situ hybridization.

## Results

### Deep sequencing of sRNAs

**Fig. 1:**
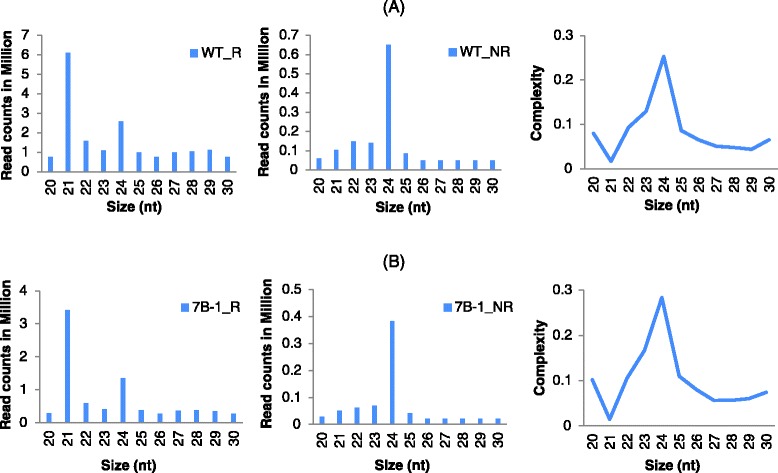
Size distribution of redundant (R) and non-redundant (NR) sRNA reads and their complexity in WT (panel **a**) and *7B-1* (panel **b**) anthers

Anthers from the flower buds at three stages of pre-meiosis, meiosis, and post-meiosis (stages 1, 2, and 3) were dissected and pooled as described by Sheoran et al., [2]. Two sRNA libraries were constructed from WT and *7B*-*1* pooled anthers and sequenced, which produced about 108 and 52 million raw reads, respectively. After adaptor trimming, reads were mapped to the tomato (cv. Heinz) genome with no mis-matches allowed (Table 1). Size class distribution of total and unique sRNAs and their complexities in each library is presented in Fig. 1. The majority of sRNAs in both *7B*-*1* and WT libraries were 21–24 nt, and formed a bi-modal size distribution typical for plant sRNAs. In general, the peak represented by 24-nt size class is greater than the 21-nt class as reported in several plant species [25, 26, 29, 39–42] with a few exceptions, as in grapevine, where the 21-nt class was more abundant than the 24-nt class in total read numbers, but not at unique read level [43], and in *Brassica juncea*, where the 21-nt class had the major peak in total and unique reads [44].

**Table 1:** Statistics of sRNA reads

Type	WT anthers		*7B*-*1* anthers	
	Total	Unique	Total	Unique
Raw reads	108,515,964		52,194,436	
Quality filtered	108,322,391		52,054,239	
Adaptor removed	45,255,262		19,253,757	
Genome matched	28,187,399	2,062,422	12,010,196	1,018,385
rRNA	1,171,580 (4.16 %)	6,480 (0.31 %)	466,773 (3.89 %)	4,796 (0.47 %)
tRNA	2,218,468 (7.87 %)	8,460 (0.41 %)	656,434 (5.47 %)	5,911 (0.58 %)
snoRNA	117,074 (0.42 %)	2,958 (0.14 %)	58,061 (0.48 %)	2,277 (0.22 %)
snRNA	107,893 (0.38 %)	8,235 (0.40 %)	46,202 (0.38 %)	5,783 (0.57 %)

In our study, the 21-nt class had a bigger peak than 24-nt class in total reads in both libraries; however, for the non-redundant distribution, the 24-nt class had the major peak in both libraries. Two key distinguishing features of sRNA libraries are the size distribution and population complexity as defined by the ratio of unique/total reads. The lower complexity of the 21-nt class in comparison to the 24-nt class in our study indicated that a relatively small number of unique reads were highly expressed in the 21-nt class, while the 24-nt class high complexity indicated the presence of more unique reads with less redundancy, which is also a typical feature of the 24-nt hcRNAs, where often are present in a more chaotic dicing pattern [45].

### Identification of known and new miRNAs

Known miRNAs from 32 known families of miRNAs were identified, which were present in both *7B*-*1* and WT libraries (Additional file 1: Table S1). The composition of miRNA families was quite similar in the two libraries, and none showed a clear *7B*-*1* or WT-specific profile. Most of the families had several members, with exception of miR171, miR393, miR398, miR403, miR530, miR858, miR4414, miR8577, miR9471, and miR9474, which were represented only by one member in both libraries (Additional file 1: Table S1). After excluding sRNA reads homologous to known miRNAs and other non-coding RNAs from Rfam v.12 database, the remaining 20-22 nt reads were used as input for miRNA prediction using the miRCat tool [46, 47]. A total of 23 putative new miRNAs were identified in the two libraries, where 11 were present only in WT, 5 in *7B*-*1*, and 7 in both libraries. The miRNA* sequences were found for two new miRNAs, which were present in both libraries and designated as miR#A and miR#B. These miRNAs formed near-perfect hairpin structures (Additional file 2: Figure S1).

### Expression analysis of miRNAs

To identify miRNAs related to anther development and regulation of male-sterility in *7B*-*1*, expressions of miRNAs were compared between *7B*-*1* and WT anther libraries. The two libraries were normalized using the reads per million (RPM) approach [48]. The differential expression analysis was conducted using the offset fold change (OFC) approach [25] and two-fold threshold was considered as a cutoff value. Among the known miRNAs, miR390, miR166, miR159 were up-regulated and miR530, miR167, miR164, miR396, miR168, miR393, and miR8006 were down-regulated in *7B*-*1* anthers (Table 2). MiR166 and miR164 had three and two iso-miRs, respectively, while the rest of differentially expressed miRNAs were present only by one member. Out of the total of 23 putative new miRNAs only two potential miRNAs, so-called mir#W and mir#M were differentially down-regulated in *7B*-*1*. None of the new miRNAs with sequenced miRNA* strands were differentially expressed in *7B*-*1* (Additional file 1: Table S2 and Table S3).

**Table 2:** List of differentially expressed miRNAs between WT and *7B*-*1* anthers

miRNA	Sequence	Read counts		Normalized expression		DE^a^	*p*-value
		WT	*7B*-*1*	WT	*7B*-*1*	Log2(WT/*7B-1*)	
miR390	AAGCTCAGGAGGGATAGCGCC	600	668	255.4	667.4	−1.3	0.003063
miR166	TCTCGGACCAGGCTTCATTCC	1731139	1537345	736984.2	1536039.9	−1.1	0.00993
miR159	TTTGGATTGAAGGGAGCTCTA	8237	7112	3506.7	7106.0	−1.0	0.016877
miR530	TGCATTTGCACCTGCACCTCC	290	50	123.5	50.0	1.0	0.022945
miR167	TGAAGCTGCCAGCATGATCTA	640	97	272.5	96.9	1.3	0.004468
miR164	TGGAGAAGCAGGGCACGTGCA	2898	466	1233.7	465.6	1.4	0.002397
miR396	TTCCACAGCTTTCTTGAACTG	2750	422	1170.7	421.6	1.4	0.002397
miR168	CCCGCCTTGCATCAACTGAAT	370	45	157.5	45.0	1.5	0.001237
miR393	ATCATGCTATCCCTTTGGACT	453	39	192.9	39.0	1.9	5.90E-05
miR8006	TAGTTTTTGGACTGCAGGGGCACC	855	34	364.0	34.0	2.8	<0.00001

^a^DE is differential expression values which were calculated as log2-fold changes of the expression. Negative and positive values mean up- and down regulation of the expression in *7B*-*1*, respectively. DE value of ±1 was considered as a cutoff value for significant changes of the expression

### RT-qPCR validation of miRNAs expression

**Fig. 2:**
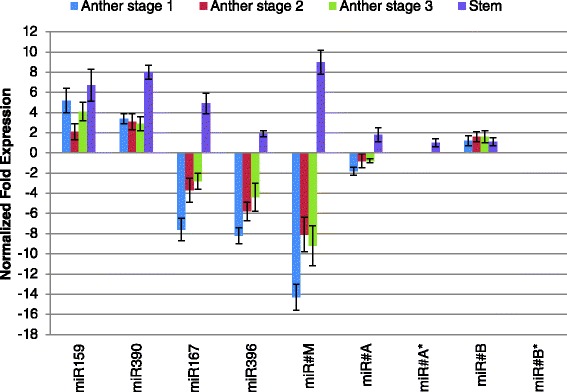
RT-qPCR validation of differentially expressed known and new miRNAs and miRNAs with miRNA*. Expression changes are presented as normalized fold changes between *7B-1* and WT reference tissue. Positive and negative values indicate up- and down-regulation of the expression, respectively. Two-fold threshold was considered as a cutoff value for significant changes in the expression. Error bars represent standard errors of three biological replicates based on DMNRT (*p* = 0.05)

Expressions of known and new miRNAs of interest were further analyzed by RT-qPCR (Fig. 2) in *7B*-*1* and WT anthers (stages 1, 2, and 3) and stem. MiR159 and miR390 were up-regulated at similar extent in all stages of *7B*-*1* anthers. MiR167 and miR396 were down-regulated in all stages of *7B*-*1* anthers; more strongly at stage 1. MiR#M was strongly down-regulated in all stages of *7B*-*1* anthers, while miR#A and miR#B were not differentially expressed and their sequenced miRNA* strands were not detected by our RT-qPCR analysis. The RT-qPCR results were in general agreement with those from sequencing data. In *7B*-*1* stem, miR159, miR390, miR167 and miR#M were all up-regulated, while miR396, miR#A and miR#B were not differentially expressed. MiR#B* was not detected by RT-qPCR. The results showed a very distinct anther- or stem-specific expression pattern of miR167, miR396, and miR#M, which suggests that they may regulate different biological processes in these tissues.

### Target prediction for known and new miRNAs

**Fig. 3:**
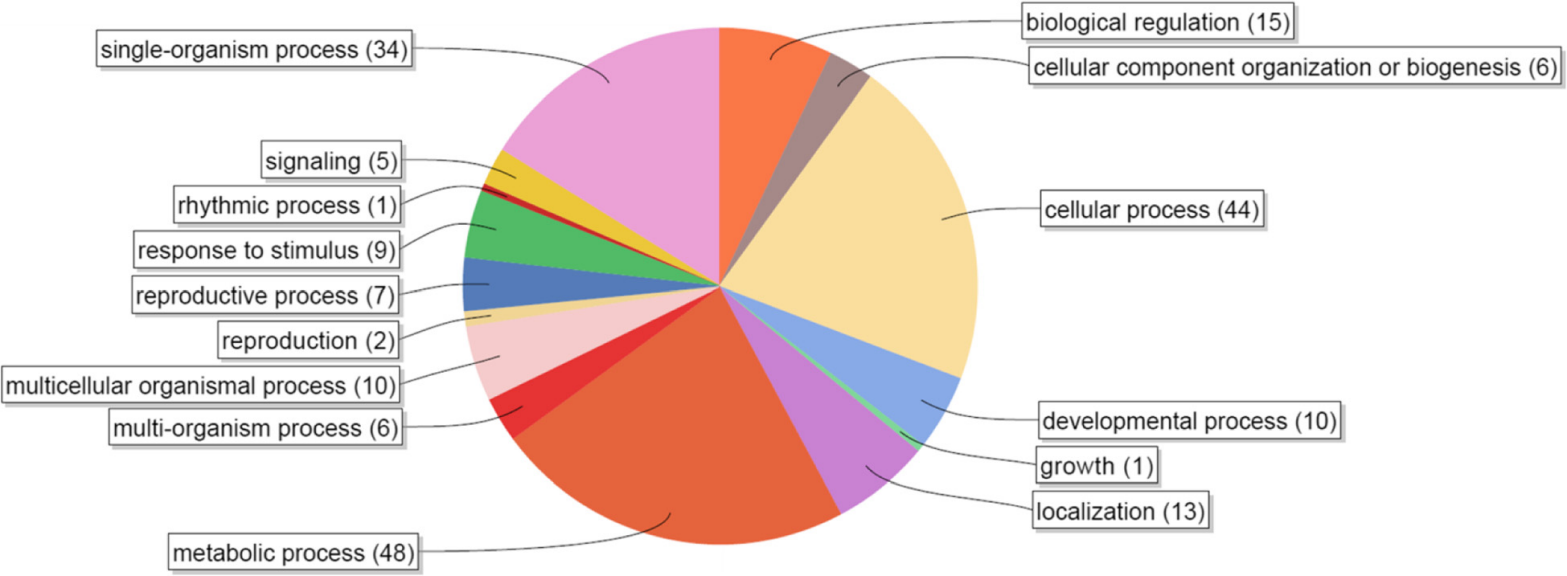
Gene ontology of miRNA targets. Predicted targets were categorized into different biological classes and numbers in the parenthesis indicate the frequency of members in each category

To better understand the biological roles of miRNAs in regulation of *7B*-*1* male-sterility, putative targets of differentially expressed known and new miRNAs were identified (Tables 3 and 4; cleavage-sites are listed in Additional file 1: Table S4). Target genes for many of known conserved miRNAs have been experimentally validated [9, 14, 17, 31, 32, 49–52]. Predicted targets for known miRNAs in our study were also in agreement with those from the literature, however a number of new putative target genes were also computationally identified, which has yet to be validated. The miRNA targets were categorized into fifteen different biological classes (Fig. 3) with the three most frequent being metabolic process, cellular process, and single-organism process. Among the targets worth mentioning (Table 3) were those, including auxin-responsive factor 8 (ARF8), ABC transporters, G protein, F-box and no apical meristem (NAM) proteins, b-ZIP, SBP-box, MAD-box and MYB transcription factors with potential roles in anther development [53–60], cystatin, receptor-like kinase, and bHLH proteins involved in tapetum development/degeneration [61–63], kinesin-like protein and GRAS transcription factor involved in meiosis regulation [64, 65], and WD-40 protein with roles in autophagy and apoptosis [66]. Among the targets of new miRNAs (Table 4), a gene encoding *pectinmethylesterase* (*PME*) *inhibitor* (*PMEI*) was identified as the putative target of miR#M. *PME* is a cell wall modifying enzyme, which catalyzes de-esterification of pectin [67]. Identification of miRNA targets with potential roles in anther development and microsporogenesis regulation in our study, suggested that these miRNAs are likely associated or involved in regulation of *7B*-*1* male-sterility.

**Table 3:** List of the predicted targets of differentially expressed known miRNAs

miRNA	Target gene	
	SGN accession no	Annotation
miR166	Solyc02g024070.2.1	Class III HD-Zip^a^
	Solyc03g006970.1.1	Subtilisin-like protease
	Solyc03g116850.2.1	Cyclic nucleotide-binding
	Solyc03g025740.2.1	Zinc finger
	Solyc07g045410.1.1	Pentatricopeptide repeat-containing protein
miR390	Solyc00g009090.2.1	Receptor-like kinase^a^
	Solyc09g091850.2.1	Beta-glucosidase
	Solyc11g016930.1.1	serine/threonine-protein kinase
miR159	Solyc01g009070.2.1	GAMYBL1
	Solyc01g102510.2.1	WD-40
	Solyc02g078670.2.1	COP1-Interacting Protein 7
	Solyc02g090160.2.1	G protein
	Solyc03g043890.2.1	Aminotransferases
	Solyc06g073640.2.1	GAMYBL2
	Solyc07g052640.2.1	Glycosyltransferase-like protein
	Solyc09g082890.1.1	Calcium-transporting ATPase 1
	Solyc10g019260.1.1	MYB39-like
	Solyc11g072060.1.1	MYB
	Solyc01g005330.2.1	microtubule associated protein Type 1
miR530	Solyc01g091550.2.1	Aspartyl protease family
	Solyc02g083460.2.1	Aspartyl protease family protein
	Solyc02g084520.2.1	Zinc finger CCCH domain-containing protein 19
	Solyc02g085520.2.1	Adenylosuccinate synthetase, chloroplastic
	Solyc02g086930.2.1	Homeobox-leucine zipper-like protein
	Solyc03g019920.1.1	Harpin-induced protein-like
	Solyc03g093230.2.1	Aquaporin
	Solyc04g009450.1.1	Ethylene-responsive transcription factor 4
	Solyc04g081070.2.1	Heat shock protein DnaJ
	Solyc06g063170.2.1	glutamate-like receptor
	Solyc07g006030.2.1	Protein TIF31 homolog
	Solyc08g007500.2.1	Pentatricopeptide repeat-containing protein
	Solyc11g062220.1.1	PHD-finger domain protein
	Solyc12g008690.1.1	Iaa-amino acid hydrolase 11
miR393	Solyc01g057310.2.1	Kinesin (Centromeric protein)-like protein
	Solyc11g006310.1.1	GEX1
	Solyc02g088800.1.1	Exocyst complex component 7
	Solyc06g068840.2.1	Copine-3
	Solyc08g081890.2.1	ABC transporter
miR396	Solyc01g066500.1.1	MAD-box transcription factor 22
	Solyc00g105750.1.1	Mutator-like transposase^a^
	Solyc00g071180.2.1	Cysteine proteinase inhibitor
	Solyc01g090270.2.1	b-ZIP transcription factor
	Solyc01g094930.2.1	CAAX prenyl protease 1
	Solyc01g110450.2.1	Aldo/keto reductase subgroup
	Solyc02g023950.2.1	Receptor like kinase
	Solyc02g083190.1.1	F-box protein
	Solyc03g058930.2.1	SPFH domain/Band 7 family protein
	Solyc03g114150.2.1	Aldehyde dehydrogenase
	Solyc03g118350.2.1	Actin-fragmin kinase
	Solyc05g053090.1.1	GRAS transcription factor^a^
	Solyc06g007320.2.1	Ubiquitin-activating enzyme E1
	Solyc06g059760.2.1	Transcriptional corepressor SEUSS
	Solyc07g019640.1.1	Transposase
	Solyc07g045480.2.1	Phytochrome F
	Solyc09g057910.2.1	N-alpha-acetyltransferase 25
	Solyc10g047270.1.1	Potassium transporter family protein
	Solyc11g006680.1.1	Pentatricopeptide repeat-containing protein
	Solyc11g020100.1.1	Cc-nbs-lrr, resistance protein
	Solyc12g013840.1.1	G-protein beta WD-40 repeat
miR164	Solyc03g115850.2.1	No Apical Meristem (NAM)^a^
	Solyc06g084350.2.1	U6 snRNA-associated Sm-like protein LSm2
	Solyc11g066150.1.1	Bifunctional polymyxin resistance protein ArnA
miR167	Solyc02g037530.2.1	Auxin response factor 8^a^
	Solyc01g010020.2.1	Flagellar calcium-binding protein
	Solyc01g086900.2.1	tRNA/rRNA methyltransferase
	Solyc01g099790.2.1	Zinc finger
	Solyc03g007790.2.1	Serine/threonine protein kinase
	Solyc03g117700.1.1	Folate/biopterin transporter
	Solyc08g065360.2.1	Glutathione-regulated potassium-efflux system protein
	Solyc08g069010.2.1	Pentatricopeptide repea
miR168	Solyc01g108250.2.1	Vacuolar import and degradation protein
	Solyc04g078130.2.1	Clathrin-coat assembly protein-like
	Solyc06g053710.2.1	Ethylene receptor
	Solyc06g060160.1.1	C2 domain-containing protein
	Solyc06g076700.1.1	Unknown protein
	Solyc09g018560.1.1	Ulp1 protease family
	Solyc09g090650.2.1	Zinc finger protein
miR8006	Solyc01g008530.2.1	Cinnamoyl CoA reductase-like protein^a^
	Solyc01g008790.2.1	Phosphoesterase family protein
	Solyc04g078250.2.1	Manganese transport protein mntH
	Solyc05g014050.2.1	Inner membrane protein
	Solyc07g049440.2.1	GDSL esterase/lipase
	Solyc08g078650.2.1	Glycosyl transferase
	Solyc09g064850.2.1	Glutathione peroxidase
	Solyc10g009520.2.1	Dihydroflavonol-4-reductase
	Solyc12g013850.1.1	Glycosyl transferase
	Solyc12g088130.1.1	BHLH transcription factor
	Solyc12g096640.1.1	RNA-binding protein

^a^indicates targets which have identified from multiple loci

**Table 4:** List of the predicted targets of new miRNAs

miRNA	Target gene	
	SGN accession no	Annotation
miR#W	Solyc01g008240.2.1	Sugar/inositol transporter
	Solyc01g015110.1.1	Ulp1 protease^a^
	Solyc01g079390.2.1	Histone-lysine N-methyltransferase MEDEA
	Solyc01g095910.1.1	Cytochrome b561/ferric reductase transmembrane
	Solyc01g103670.2.1	Alpha/beta hydrolase fold-1 domain-containing protein
	Solyc01g103800.2.1	Ribosomal protein S12e
	Solyc01g104990.2.1	Trimethylguanosine synthase
	Solyc02g022870.2.1	EMB1611/MEE22
	Solyc02g069410.2.1	HAD-superfamily hydrolase subfamily IA variant 3
	Solyc02g069740.2.1	Jumonji transcription facto
	Solyc02g092780.1.1	Tetratricopeptide-like helical
	Solyc03g078240.1.1	UDP-glucosyltransferase
	Solyc03g115860.2.1	Endoplasmic reticulum membrane protein
	Solyc04g074660.1.1	Folate-sensitive fragile site protein Fra10Ac1
	Solyc04g079240.2.1	Patatin
	Solyc05g007850.1.1	Tir-nbs-lrr, resistance protein
	Solyc06g016750.2.1	CBF transcription factor
	Solyc06g065180.2.1	SLL1 protein
	Solyc06g083070.2.1	Actin filament bundling protein
	Solyc08g014530.1.1	Subtilisin-like protein
	Solyc08g069000.2.1	Zinc transport protein zntB
	Solyc08g081290.2.1	ARID/BRIGHT DNA-binding protein
	Solyc09g007370.2.1	Ribonuclease P protein subunit p29
	Solyc09g083200.2.1	Nod factor receptor protein
	Solyc09g089550.2.1	Zinc finger-homeodomain protein 2
	Solyc10g084410.1.1	Phosphatase 2C family protein
	Solyc12g027580.1.1	Exportin 4
	Solyc12g077660.1.1	Nucleosome assembly protein (NAP)
miR#M	Solyc03g112990.1.1	Pectinesterase inhibitor
miR#A	Solyc03g119580.1.1	Ethylene-responsive transcription factor 4
	Solyc10g078230.1.1	Cytochrome P450
miR#B	Solyc01g044350.2.1	Zinc finger (Ran-binding) family protein
	Solyc02g089170.2.1	Alpha-1,4-glucan-protein synthase
	Solyc03g113250.2.1	Nitrate transporter
	Solyc04g007260.2.1	Thioesterase superfamily
	Solyc04g009620.2.1	Chorismate synthase
	Solyc04g071160.2.1	b-ZIP transcription factor
	Solyc05g054890.2.1	ABC transporter G family member 1
	Solyc06g084100.2.1	Protein phosphatase 2C
	Solyc07g005390.2.1	Aldehyde dehydrogenase
	Solyc07g007230.2.1	Heat shock protein
	Solyc07g015870.2.1	Polygalacturonase 1
	Solyc08g069030.2.1	Tetrapyrrole biosynthesis
	Solyc09g074100.2.1	tRNA methyl transferase-like
	Solyc09g091230.2.1	Glycosyl transferase

^a^indicates targets which have identified from multiple loci

### Identification of ta-siRNAs and prediction of their targets

*TAS* loci and their associated ta-siRNAs were predicted based on the phased 21 nt sRNAs characteristic of ta-siRNA loci [46]. Ta-siRNAs with abundance below 10 reads were excluded from the analysis. Twenty-five ta-siRNAs from WT, 7 from *7B*-*1* and 7 common in both libraries were identified and their target genes were predicted (Additional file 1: Table S5). As these ta-siRNAs had generally low abundances, those identified only in WT or *7B*-*1* could not be considered as WT- or *7B*-*1*-specific and were not further analyzed. Instead, we focused on those which have been found in both libraries; however none were differentially expressed (Additional file 1: Table S5). Ta-siRNAs with phased expressions matching to the tomato *TAS3* (Solyc01g058100.2.1) were also identified (Additional file 1: Table S6 and Table S7), however *TAS3*-derived 5′D7(+) and 5′D8(+) tasi*ARFs* were not differentially regulated between the two libraries. The results suggested that ta-siRNAs were not likely associated or involved in regulation of male-sterility in *7B*-*1* as they were not differentially regulated.

### 5′-RACE validation of miRNA and ta-siRNA targets

**Fig. 4:**
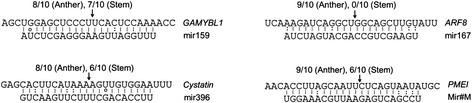
5′- RACE validation of miRNA target genes in *7B*-*1* anther and stem. Gene transcripts are in 5′-3′ and miRNAs in 3′-5′directions. The arrows indicate the cleavage sites of target mRNA and numbers above them indicate frequency (out of 10) of sequences found at the exact miRNAs cleavage sites. Watson-Crick pairing (*vertical dashes*), G-U wobble pairing (*circles*), and other mismatches (:) are indicated

The predicated targets of interest were validated in *7B*-*1* anthers (stage 1; where changes of the expression were strongest) and stem using 5′-RACE. Sequence analysis showed (Fig. 4) that the 5′ ends for most of the cleaved mRNA fragments corresponded to nucleotide complementary to the 10^th^ nucleotide of the corresponding miRNA. MiR159 and miR319 could both cleave *MYB* transcripts [32]; however sequence analysis of the cleavage products showed that *MYB* cleavage in anthers and stem was directed by miR159, not miR319. Cleavage products of miR159 and miR319 can be readily distinguished as they differ in one base. Cleavage products of *ARF8*, target of miR167, were identified from anther, but not stem. Among the predicted targets of miR396, cleavage products of *cystatin* were identified in both anther and stem libraries, but not *MAD*-*box*. Cleavage products of *PMEI*, predicted target of miR#M, were also identified in both anther and stem libraries. Cleavage products of *ARF2*, *3* and *4*, targets of D7 and D8 tasi*ARFs*, were identified from both anther and stem libraries (Additional file 2: Figure S2). These results showed that the above mentioned miRNAs, including the newly identified miR#M as well as tasi*ARFs* were functionally active in *7B*-*1* anther and stem, where directed the cleavage of their predicted target transcripts. Furthermore, it provided experimental evidence to support our target predictions.

### RT-qPCR analysis of miRNA and ta-siRNA targets

**Fig. 5:**
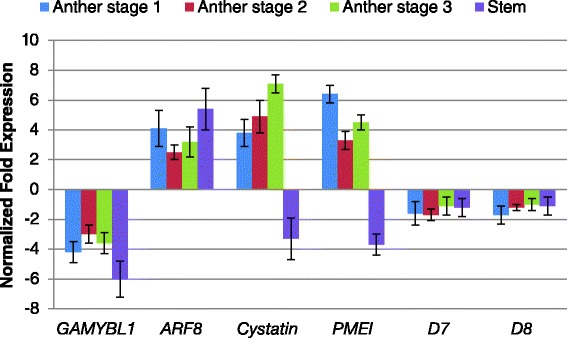
RT-qPCR validation of miRNA target genes and Tasi*ARFs* in *7B*-*1* anthers and stem. Expression changes are presented as normalized fold changes between *7B*-*1* and WT reference tissue. Positive and negative values indicate up- and down-regulation of the expression, respectively. Two-fold threshold was considered as a cutoff value for significant changes in the expression. Error bars represent standard errors of three biological replicates based on DMNRT (*p* = 0.05)

Figure 5 shows RT-qPCR analysis of miRNA targets of interest and Tasi*ARFs* in *7B*-*1* anthers and stem. *GAMYBL1* was down-regulated in anthers of all stages and more strongly in stem. *ARF8* was up-regulated in anthers of all stages and stem. *Cystatin* and *PMEI* were both up-regulated in anthers of all stages, but down-regulated in stem. Tasi*ARFs* (D7 and D8) were not differentially expressed in anthers and stem. *ARF2*/*3*/*4* were not differentially expressed in anthers, but up-regulated in stem (Additional file 2: Figure S3). Analysis showed a consistent negative correlation between miRNAs expression and accumulation level of their targets, except for miR390-*TAS3*-tasi*ARFs* (in anthers and stem), and miR167-*ARF8* (in stem). In addition to miRNA and their targets, expression of two cell wall modifying enzymes, *cysteine protease* and *polygalacturonase*, were also analyzed in *7B*-*1* anthers and stem (Additional file 2: Figure S4). *Cysteine protease* was down-regulated in anthers, more strongly at stage 3, while up-regulated in stem. *Polygalacturonase* was strongly down-regulated in anthers at stage 2, but not differentially regulated at stage 3, nor in stem.

### In situ hybridization

**Fig. 6:**
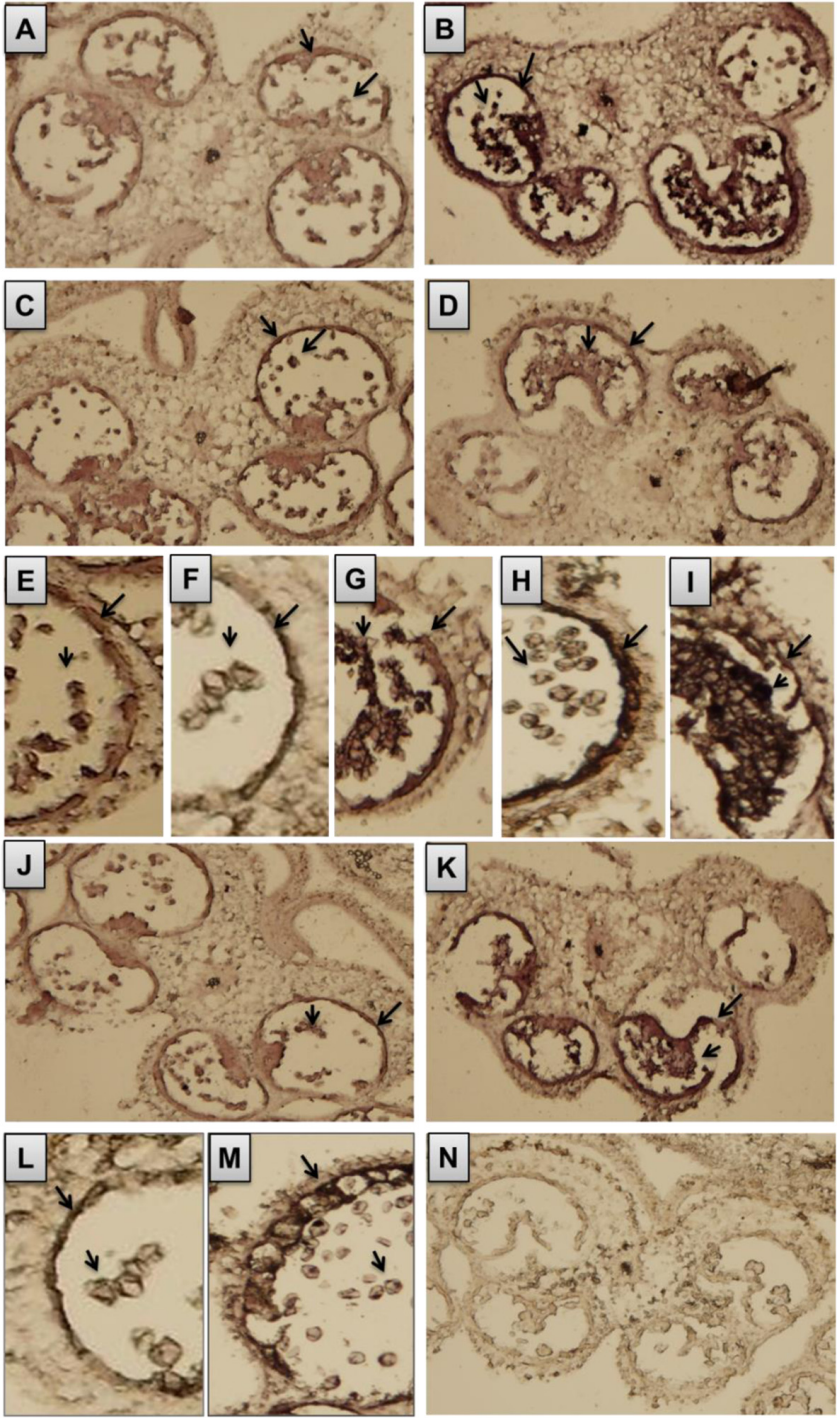
In situ localization of miR159, *GAMYBL1*, *PMEI* and *cystatin*. **a** and **b** localization of miR159 in WT and *7B*-*1* anthers, respectively. **c** and **d** *GAMYBL1* in WT and *7B*-*1* anthers, respectively. **e** and **f** *PMEI* in WT anthers at late meiotic and binucleate microspores stages, respectively. **g**, **h**, and **i** *PMEI* in *7B*-*1* anthers at late meiotic stage, free binucleate microspores, and arrested binucleate microspores, respectively. **j** and **k** *cystatin* in WT and *7B*-*1* anthers, respectively. **l** and **m** *cystatin* in WT and *7B*-*1* anthers at binucleate microspores stage, respectively. **n** negative control, where a murine miR122a-specific probe was used. Arrows indicate the localization sites

Figure 6 shows in situ localization of miR159, *GAMYBL1*, *PMEI* and *cystatin* in WT and *7B*-*1* anthers in the late meiotic (where still some tetrads could be seen together with the newly formed microspores) and in binucleate microspores stages as for the cases of *PMEI* and *cystatin*. MiR159, *GAMYBL1* and *cystatin* were all predominantly expressed in tapetum, tetrads and free microspores in WT and more strongly in *7B*-*1* as the case of miR159 and *cystatin*. Panels L and M of the figure show WT and *7B*-*1* anthers, respectively at binucleate microspores stage, where tapetum was degenerated in WT, but not degenerated and vacuolated in *7B*-*1. Cystatin* was strongly expressed in vacuolated tapetal cells in *7B*-*1* (Panel M). *PMEI* was strongly expressed in tapetum, tetrads and free microspores in *7B*-*1* anthers, but its expression was mainly restricted to tapetum at binucleate microspores stage (Panel H). As mentioned earlier, in some of the *7B*-*1* anthers, microspores were not separated and remained attached after meiosis. *PMEI* was strongly expressed in arrested binucleate microspores in *7B*-*1* (Panel I). No detectable hybridization signal was observed for the murine miR122a probe, which served as a negative control.

## Discussion

The main goal of our study was to investigate the sRNA profiles, particularly miRNAs, between the *7B*-*1* mutant and WT anthers and to identify differentially expressed miRNAs and their targets with potential roles in anther development and regulation of male-sterility in *7B*-*1*. Analysis showed that the overall size distribution and population complexity of sRNAs were quite similar between the *7B*-*1* and WT anther libraries. Composition of sRNAs could indicate the roles and activity level of different categories of sRNAs in a particular tissue or species or associated biogenetic machineries. In our study, the 21-nt class had a larger peak than the 24-nt class in total reads in both libraries; however, in non-redundant format, the 24-nt class had the major peak in both libraries. The 24-nt class was also found to be the major peak in anthers of a male-sterile cotton [28]. Higher abundance of the 21-nt class in total reads in anthers of *7B*-*1* and WT in our study, could suggest a more active roles for this class of sRNAs and an intensified contribution from miRNA biogenesis pathways that produce the more precisely defined sRNA species at those stages compared to those of other crops having the 24-nt class with higher redundancy.

**Fig. 7:**
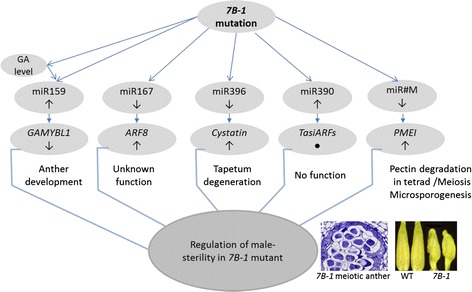
Schematic presentation of miRNA-target pairs and their role in regulation of anther development and male-sterility in *7B*-*1* mutant. Arrows below miRNAs and miRNA targets show up- and down-regulation of the expression. “●” indicates no change in the expression. Pictures of *7B*-*1* anthers are adopted from Sheoran et al., [2]

Even though there is no direct evidence that any miRNAs are causative genes for male-sterility in plants, we hypothesized that differential expression of miRNAs could be associated with the regulation of male-sterility in *7B*-*1*. Thirty-two families of known miRNAs were identified in both *7B*-*1* and WT anther libraries. Three and seven miRNA families were up and down-regulated, respectively in *7B*-*1* anthers. Out of the 23 putative new miRNAs only two, so-called miR#W and miR#M, were differentially expressed in *7B*-*1* anther. Expressions of miRNAs of interest were further analyzed at different stages of *7B*-*1* anthers and stem. In general, changes of the expression were strongest in anthers at stage 1, where the MMCs were about to undergo the meiosis. MiR167, miR396, and miR#M had distinctively different expression patterns in anthers and stem, which strongly suggested that these miRNAs regulate different biological processes in these tissues. The predicted targets of differentially expressed miRNAs were categorized into fifteen different biological classes, with metabolic process, cellular process, and single-organism process being the three most frequent classes. Among these miRNAs, miR159, miR167, miR396, miR390 and miR#M were those of particular interest as their targets had potential roles in anther development, microsporogenesis and production of ta-siRNAs as the case of miR390. A schematic presentation of miRNA-target pairs and their role in regulation of male-sterility in *7B*-*1* mutant is illustrated in Fig. 7. 5′-RACE validation of these miRNA targets in *7B*-*1* anther and stem showed that they were active and directed the cleavage of their targets in these tissues, except for miR167 in *7B*-*1* stem.

MiR159 targets several *GAMYBs* [55, 68, 69], among them *AtMYB33*, *AtMYB65* act redundantly in regulation of anther development [55]. Overexpression of miR159 disrupted anther development and led to male-sterility in *Arabidopsis thaliana* [36]. 5′-RACE analysis in our study showed that miR159 directed the cleavage of *GAMYBL1* transcripts out of the 4 predicted *MYB* targets in anther and stem. *OsGAMYBL1* and *2* are expressed in rice anthers and regulated by miR159, however functions of these gene are not characterized [69]. *LeGAMYBL1* plays a role in seed development in tomato [70], but it is not functionally characterized with respect to anther development. Analysis showed that the expression of *GAMYBL1* was negatively correlated with the miR159 level in *7B*-*1* during anther development and in stem. In situ hybridization analysis in *7B*-*1* anthers also showed that miR159 and *GAMYBL1* were both expressed mainly in tapetum, tetrads and microspores. MiR159 and *GAMYBL1* were down- and up-regulated, respectively in GA-treated *7B*-*1* anthers (Additional file 2: Figure S5). These observations indicated that miR159-*GAMYBL1* cleavage cascade and GA level in flower buds are tightly linked to the regulation of anther development and male-sterility in *7B*-*1*.

Most of the elongated mutants have been reported as GA-overproducers [71–73]; however elongated stem of *7B*-*1* had lower levels of GAs compared to WT in long days [4]. Several *MYBs* have been identified, which positively regulate stem elongation in light via interaction with phytochromes and/or regulation of light-responsive genes [74, 75]. Down-regulation of *GAMYBL1* in *7B*-*1* stem is likely associated with the elongated stem phenotype, however functional analysis are needed to understand the actual function of this gene.

MiR167 cleaves *ARF6*/8 transcripts [76]; however we only identified *ARF8* in our target prediction, which was also validated by 5′-RACE in *7B*-*1* anther. Tomato overexpressing miR167 and *arf6*-*arf8* double-null mutant of *Arabidopsis thaliana* both had flowers with severe defects associated with female and male-sterilities, respectively [76, 77]. Therefore, proper regulation of miR167-*ARF6*/8 is required for normal female and male organ development. RT-qPCR analysis showed strong down-regulation of miR167 in *7B*-*1* anthers, which was negatively correlated with regulation of *ARF8* in anthers. Although differential regulation of miR167-*ARF8* cleavage cascade in *7B*-*1* anthers could be linked and due to *7B*-*1* mutation, its actual function with respect to anther development and male-sterility in *7B*-*1* remains to be further characterized. In *7B*-*1* stem, neither the *ARF8* cleavage products were found nor the *ARF8* expression was correlated with miR167 level. Tomato overexpressing miR167 had lower levels of *ARF6*/8 transcripts and a shorter hypocotyl [76], while other researchers reported that light-grown *Arabidopsis thaliana arf8*-*1* mutant had elongated hypocotyl [78]. Although up-regulation of miR167 and *ARF8* in *7B*-*1* stem could be independently associated and/or affected by *7B*-*1* mutation, understanding their functions with respect to anther development and male-sterility in *7B*-*1* requires further functional analysis.

To the best of our knowledge no reports have yet documented ta-siRNAs in regulation of male-sterility in plants. Ta-siRNAs including *TAS3*-derived tasi*ARFs* were not differentially expressed between *7B*-*1* and WT; however miR390 was up-regulated in *7B*-*1* anther and stem. This suggested that although miR390 expression was affected by *7B*-*1* mutation, ta-siRNA biogenesis and tasi*ARFs*-guided regulation of *ARF2*/*3*/*4* were not affected by *7B*-*1* mutation nor related to anther development in *7B*-*1*. Nevertheless, 5′-RACE analysis showed that tasi*ARFs* are active in *7B*-*1* anther and stem, where directed the cleavage of *ARF2*/*3*/*4* transcripts. Target genes of *ARF2*/*3*/*4* are largely unknown, thus the mechanisms linking these *ARFs* to a context-specific role in regulation of *7B*-*1* stem growth if any are poorly understood.

Our cytological studies showed (Additional file 2: Figure S6; unpublished data) that anther development in *7B*-*1* (between anther lobes and/or among anthers) was not synchronized in contrast to the WT. We observed that MMCs did undergo meiosis and formed tetrads despite the previous report suggested a breakdown of meiosis in MMCs [2]. More importantly, we found that in some anther lobes, the newly formed microspores were not separated, while in others they formed free microspores. In *qrt1* and *qrt2* mutants of *Arabidopsis thaliana*, microspores failed to separate from tetrads as pectin was not degraded in the primary cell wall [79]. *PMEI* (miR#M target) was up-regulated in *7B*-*1* anthers, where the expression was mainly localized in tapetum, tetrads and microspores. *PMEI* was strongly expressed in the arrested binucleate microspores, compared to the free binucleate microspores, where a basal expression was detected. *PMEs* and *PMEIs* are key regulators of pollen cell wall and pollen tube development [79–81]. Up-regulation of *PMEI* in *7B*-*1* anthers may have suppressed the *PME* activity and impaired the subsequent enzymatic degradation of pectin in the primary cell walls around tetrads. In *qrt3*, mutation in a gene encoding a *polygalacturonase* (pectin modifying enzyme) impaired the degradation of pectin in the primary cell wall, similar to *qrt1* and *qrt2* mutants [82]. *Polygalacturonase* was also down-regulated in *7B*-*1* anthers in our study. Based on our cytological and transcriptional data, we suggest a potential association between the regulation of miR#M-*PMEI* and *polygalacturonase* and anther development in *7B*-*1* as inhibition of *PME* and *polygalacturonase* activities could have disrupted pectin degradation and proper separation of tetrads and/or tapetum degeneration. However, it could not be the cause of male-sterility in *7B*-*1* as some of the anthers were still able to produce mature pollens.

*Arabidopsis thaliana* overexpressing *PME* showed reduced cell elongation in hypocotyl [83]. Al-Qsous et al., [84] suggested that higher level of *PME* transcript in elongated hypocotyl of flax was associated with cell wall stiffening. Overexpression of a *polygalacturonase* gene, *PGX1*, enhanced hypocotyl elongation in etiolated *Arabidopsis thaliana* [85]. Understating the function of *PME* and *polygalacturonase* in regulation of stem elongation in *7B*-*1* if they have indeed altered the pectin level and how they are connected to the cell expansion requires further analysis.

While conserved miRNAs regulate expression of the genes involved in basic developmental processes, the non-conserved or new miRNAs may be involved in the development of traits that are specific for certain species. New miRNAs are being continually identified from different species, including tomato [24, 25]. In addition to miR#M, we identified a number of new miRNAs, two with sequenced miRNAs*, which could form a near prefect hairpin structures.

Sheoran et al., [2] identified a number of differentially expressed proteins between *7B*-*1* and WT anthers, where cystatin showed the strongest up-regulation of all in *7B*-*1* anthers. The cystatin inhibitory activity was also higher in *7B*-*1* anthers relative to WT [2]. In plants, *cysteine proteases* act as key regulators of programmed cell death in tapetal cells and pollen development, and suppression of their expression has often resulted in delay or failure in tapetum degeneration, pollen abortion and male-sterility [62, 86–88]. MiR396 directed the cleavage of *cystatin* in *7B*-*1* anthers and stem. *Cystatin* was up-regulated and *cysteine protease* was strongly down-regulated as a result in *7B*-*1* anthers, with a pattern closely correlated to the tapetum degeneration during anther development. These observations provided a strong evidence to suggest that suppression of *cysteine protease* could have caused a delay or failure in tapetum degeneration, thereby affecting microsporogenesis and anther development in *7B*-*1*. The length of stem in *7B*-*1* is tightly correlated with the length of epidermal cells [6]. Minic et al., [89] identified several *cysteine proteases* in developing stems of *Arabidopsis thaliana*, suggested to be involved in cell expansion and secondary wall formation. Higher expression of *cysteine protease* in our study could be associated with the higher cell expansion rate and longer epidermis cells in *7B*-*1* stem. Overall in our study, we found that miRNA-mediated regulation of gene expression was perturbed in the *7B*-*1* mutant line. The findings strongly supported that miR159-*GAMYBL1*, miR396-*cystatin* and miR#M-*PMEI* cleavage cascades were tightly connected to the regulation of microsporogenesis and anther development in *7B*-*1*. Stem elongation in *7B*-*1* may be regulated via a complex web of molecular components and interaction between miRNAs and hormones, which requires further functional studies to be understood. A number of new miRNAs were also identified for the first time from tomato, which provides new opportunities to study the unexplored functions of these miRNAs in tomato. Our data could be used as a benchmark for future studies of the molecular mechanisms of male-sterility in other crops.

## Conclusion

Using sRNA sequencing, we studied miRNA profiles during anther development between *7B*-*1* mutant and WT. Comparison of expression of miRNAs and their targets between *7B*-*1* and WT and in situ localization analysis suggested potential involvement of several miRNA-target pairs in regulation of anther development and male-sterility in *7B*-*1*. In addition a number of new miRNAs were identified and validated for the first time from tomato.

### Availability of supporting data

The sequences could be found in NCBI database under accession number GSE65788.

## Methods

### Plant materials

The *7B*-*1* mutant and WT seedlings (*Solanum lycopersicum* L., cv. Rutgers) were grown in temperature controlled growth chamber set for long days condition (16/8 h light/dark). Flower buds of different sizes smaller, equal and bigger than 4-5 mm (referred as stages 1, 2, and 3 hereafter) were collected and stamens were dissected under a microscope. It should be noted that stamens at these stages were mostly consisted of anthers, with little or on filament growth. Stages of flower buds were based on those described by Sheoran et al., [2] and also further confirmed by analysis of anther squashes. Flower buds at stage 1 represents pre-meiotic anthers, stage 2 is where tetrads are formed in WT anthers (meiotic anthers), but meiosis breaks down in MMCs in *7B*-*1* [2]. Stage 3 represents post-meiotic anthers. Stems from three-month old seedlings were used for qPCR and 5′-RACE analysis.

### RNA analysis

Total RNA was extracted using the RNeasy Plant Mini Kit (Qiagen) from *7B*-*1* and WT anthers of different stages and pooled separately in equimolar ratio. Two sRNA libraries were constructed using the TruSeq Small RNA Sample Preparation Kit (Illumina). In brief, sRNA fractions of 18–40 nt were isolated from 15 % denaturing polyacrylamide gels, ligated to the 5′ and 3′ TruSeq adaptors and then converted to DNA by RT-PCR following the kit protocol. The final PCR products were purified from the gel and sequenced using Illumina Hiseq2000 platform (Illumina)

### Sequence analysis

Adaptor sequences trimmed and reads were mapped (no mismatch allowed) to the tomato genome ITAG v2.5 Release using PatMaN [90] and a customized Perl script. Reads mapping to tomato repeats, transposons, intron, CDs, and promoter regions were identified. Sequences were searched against rRNA, tRNA, snRNAs and snoRNAs from Rfam v.12 and NCBI nt/nr databases. Known miRNAs were identified using miRprof [47], (available from the UEA sRNA workbench; http://srna-tools.cmp.uea.ac.uk/) allowing two mismatches with the mature miRNAs in miRBase database release 21 [91]. New miRNAs were predicted using miRCat (UEA sRNA toolkit), and their secondary structures were analyzed using a RNA hairpin folding and annotation tool (UEA sRNA toolkit). The parameters for miRcat included a minimum of 17 nucleotides paired, a maximum of two gaps between the miRNA and miRNA*, a maximum of 10 genomic hits, a minimum hairpin length of 70 nt, a minimum GC content of 20 %, a maximum of 60 % unpaired nucleotides and a strand bias of 80 % of sequences on one strand (regardless of the strand). These parameters were determined empirically on plant datasets [46, 47]. The significance testing for the secondary structure was conducted with RandFold [92].

Targets of miRNAs were predicted (allowing 4 mismatches) using the tomato ITAG cDNA v2.5. Expression values of miRNAs were normalized against the total number of genome-mapping reads [48] and changes in the expression were calculated as offset-fold change as described in Mohorianu et al., [25]. The p-values were calculated based on the standardized distribution of differential expression for all genome-matching sRNAs. *TAS* loci were predicted based on phased 21-nt sRNAs characteristic of ta-siRNA loci using a TASI prediction tool (UEA sRNA toolkit). Gene ontologies were assigned using the Blast2go tool (http://www.blast2go.com/b2ghome).

### Quantitative PCR

Expressions of miRNAs and ta-siRNAs were validated using the Mir-X™ miRNA First-Strand Synthesis and SYBR® RT-qPCR kit (Clontech). In a single reaction, sRNA molecules were polyadenylated and reverse transcribed using poly(A) polymerase and SMART™ MMLV Reverse Transcriptase provided by the kit. List of miRNA and ta-siRNA forward primers is provided in Additional file 1: Table S8. U6 small nuclear RNA was used as a reference for data normalization. QPCR conditions were set at 95 °C for 10 s, followed by 40 cycles of 95 °C for 5 s, and annealing/extension at 60 °C for 20 s. Changes of expressions were calculated as normalized fold ratios using the ΔΔCT method [93]. QPCR validations of miRNA target genes were carried out using the SensiFAST SYBR Lo-ROX kit (Bioline). First-strand cDNAs were synthesized using the PrimeScript First Strand cDNA Synthesis kit (Takara). Gene-specific primers spanning the miRNA cleavage sites were designed and listed in Additional file 1: Table S8. Housekeeping *α*-*tubulin* and *CAC* genes were used as reference genes for data normalization (data were shown only for *α*-*tubulin*). PCR conditions were set at 95 °C for 2 min, followed by 40 cycles of 95 °C for 5 s, and annealing/extension at 60 °C for 20 s.

### 5′-RACE analysis

miRNA targets of interest were functionally validated using the GeneRacer kit (Invitrogen) through RNA ligase-mediated rapid amplification of 5′ cDNA ends (RLM-5′ RACE). In brief, 5 μg of total RNA was used to purify the mRNA. The 5′ RNA adaptor was ligated to the degraded mRNA with a 5′ free phosphoric acid by T4 RNA ligase, followed by a reverse transcription reaction. Subsequently, 1 μl of 10X diluted reverse transcription product was used to amplify the 5′ end of the corresponding targets using the 5′ GeneRacer and 3′ gene-specific primers. Final PCR products were analyzed by gel electrophoresis and cloned into the pCR®4-TOPO vector (Invitrogen). Ten different colonies were subjected to sequence analysis. The reverse gene-specific primers are listed in Additional file 1: Table S9.

### In situ hybridization

Flower buds were fixed overnight in 4 % paraformaldehyde, then dehydrated using a graded ethanol series (50, 70, 95 and 100 %) and embedded in Paraplast® Plus™ chips. Transverse sections of 8 μM thick were prepared from the embedded blocks using a Leica Ultracut R ultramicrotome (Leica Bensheim, Germany). In situ hybridization was carried out following the protocol described by Javelle and Timmermans, [94]. 5′-end DIG-labeled oligo-probes (Additional file 1: Table S10) with sequences complementary to miR159, *GAMYBL1*, *PMEI*, *cystatin*, and murine miR122a (as a negative control) were synthesized by Eastport (Eastport, Czech Republic). Probe concentration of 10 nM and hybridization temperature of 50 °C were experimentally identified as optimums. In situ localization signals were detected in colorimetric reactions using DIG-specific antibody coupled to alkaline phosphatase.

### Experimental design and statistical analysis

The experiments were arranged in a completely randomized design with three biological replications. Data were subjected to analysis of the variance (ANOVA) and duncan new multiple range test (DNMRT *p* = 0.05) for comparison of the means using the SAS software version 9.2.

## Additional files

Additional file 1: Table S1. List of the identified known miRNAs in WT and *7B*-*1* anthers. **Table S2.** List of the new miRNAs identified from WT anthers. **Table S3.** List of the new miRNAs identified from *7B*-*1* anthers. **Table S4**. List of miRNA-target cleavage sites. **Table S5.** List of tasiRNAs and their predicated target genes. **Table S6.** List of the identified *TAS3*-derived tasiRNAs from WT library. **Table S7.** List of the identified *TAS3*-derived tasiRNAs from *7B*-*1* library. **Table S8.** List of the primers used for RT-qPCR analysis. **Table S9.** List of the primers used for 5′-RACE analysis. **Table S10.** List of the DIG-labeled oligo-probes used for in situ hybridization. (DOCX 676 kb) Additional file 2: Figure S1. Hairpin structures of new miRNA precursors. **Figure S2.** 5′- RACE validation of tasi*ARFs* target genes in *7B*-*1* anther and stem. **Figure S3.** RT-qPCR validation of tasi*ARFs* target genes in *7B*-*1* anther and stem. **Figure S4.** RT-qPCR validation of *cysteine protease* and *polygalacturonase* in *7B*-*1* anthers and stem. **Figure S5.** RT-qPCR validation of miR159 and *GAMYBL1* in GA-treated *7B*-*1* anthers. **Figure S6.** Cytological study of anther development in *7B*-*1* and WT. (DOCX 4243 kb)
