# Supplementary material for: Identification of miRNAs with potential roles in regulation of anther development and male-sterility in 7B-1 male-sterile tomato mutant
Source: BMC Genomics. 2015 Oct 28;16:878. doi: 10.1186/s12864-015-2077-0 (PMC4625851; doi:10.1186/s12864-015-2077-0)
Supplement: Additional file 1: Table S1. — List of the identified known miRNAs in WT and 7B-1 anthers. Table S2. List of the new miRNAs identified from WT anthers. Table S3. List of the new miRNAs identified from 7B-1 anthers. Table S4. List of miRNA-target cleavage sites. Table S5. List of tasiRNAs and their predicated target genes. Table S6. List of the identified TAS3-derived tasiRNAs from WT library. Table S7. List of the identified TAS3-derived tasiRNAs from 7B-1 library. Table S8. List of the primers used for RT-qPCR analysis. Table S9. List of the primers used for 5′-RACE analysis. Table S10. List of the DIG-labeled oligo-probes used for in situ hybridization. (DOCX 676 kb) [file 12864_2015_2077_MOESM1_ESM.docx]

**Table S1.** List of the identified known miRNAs in WT and *7B-1* anthers

| **Chr** | **Start** | **Stop** | **Strand** | **Sequence** | **Annotation** | **Normalized expression** | |
| --- | --- | --- | --- | --- | --- | --- | --- |
|  |  |  |  |  |  | **WT** | ***7B-1*** |
| SL2.40ch00 | 12537520 | 12537540 | + | TTCCACAGCTTTCTTGAACTG | ath-miR396a-5p | 1170.7 | 421.6 |
| SL2.40ch00 | 17038754 | 17038774 | - | CTGAAGTGTTTGGGGGAACTC | ath-miR395a | 35.8 | 23.0 |
| SL2.40ch01 | 2830384 | 2830404 | - | TTTTTGGACGGCAGGGGCACC | stu-miR8006-5p | 44.7 | 21.0 |
| SL2.40ch01 | 2830384 | 2830406 | - | AGTTTTTGGACGGCAGGGGCACC | stu-miR8006-5p | 44.3 | 30.0 |
| SL2.40ch01 | 2830384 | 2830407 | - | TAGTTTTTGGACGGCAGGGGCACC | stu-miR8006-5p | 37.9 | 30.0 |
| SL2.40ch01 | 3242424 | 3242447 | + | TAGTTTTTGGACTGCAGGGGCACC | stu-miR8006-5p | 364.0 | 34.0 |
| SL2.40ch01 | 70879819 | 70879839 | + | CTAGATTCACGCACAAGCTCG | ath-miR403-3p | 37.0 | 24.0 |
| SL2.40ch01 | 79167051 | 79167071 | + | GGAATGTTGTCTGGCTCGAGG | ath-miR165a-5p | 525.8 | 431.6 |
| SL2.40ch01 | 79167139 | 79167159 | + | CGTCGGACCAGGCTTCATTCC | gma-miR166h-3p | 52.8 | 112.9 |
| SL2.40ch01 | 79167140 | 79167160 | + | GTCGGACCAGGCTTCATTCCC | ppt-miR166j | 25.5 | 44.0 |
| SL2.40ch01 | 79167141 | 79167161 | + | TCGGACCAGGCTTCATTCCCC | ath-miR165a-3p | 1249274.0 | 1347676.9 |
| SL2.40ch01 | 79871316 | 79871336 | - | AAAAAGATGCAGGACTAGACC | sly-miR9476-3p | 56.6 | 48.0 |
| SL2.40ch01 | 79871367 | 79871387 | + | AAAAAGATGCAGGACTAGACC | sly-miR9476-3p | 56.6 | 48.0 |
| SL2.40ch01 | 81732061 | 81732081 | + | TGGAGAAGCAGGGCACGTGCA | ath-miR164a | 1233.7 | 465.6 |
| SL2.40ch01 | 82801319 | 82801340 | - | TGCACTGCCTCTTCCCTGGCTC | smo-miR408 | 33.6 | 32.0 |
| SL2.40ch01 | 82801320 | 82801340 | - | TGCACTGCCTCTTCCCTGGCT | ppt-miR408b | 303.1 | 601.5 |
| SL2.40ch01 | 84381907 | 84381928 | - | TCGGACCAGGCTTCATTCCTCT | ctr-miR166 | 75.8 | 117.9 |
| SL2.40ch01 | 84381908 | 84381928 | - | TCGGACCAGGCTTCATTCCTC | ath-miR165a-3p | 35012.7 | 46459.5 |
| SL2.40ch01 | 84381910 | 84381930 | - | CGTCGGACCAGGCTTCATTCC | gma-miR166h-3p | 52.8 | 112.9 |
| SL2.40ch01 | 84382032 | 84382052 | - | GGGATGTTGTCTGGCTCGACA | sly-miR166c-5p | 561.5 | 378.7 |
| SL2.40ch02 | 16516665 | 16516685 | - | TGCCTGGCTCCCTGTATGCCA | ath-miR160a-5p | 79.6 | 164.9 |
| SL2.40ch02 | 27508523 | 27508543 | - | CTGAAGTGTTTGGGGGAACTC | ath-miR395a | 35.8 | 23.0 |
| SL2.40ch02 | 27508705 | 27508725 | - | CTGAAGTGTTTGGGGGAACTC | ath-miR395a | 35.8 | 23.0 |
| SL2.40ch02 | 27526675 | 27526695 | - | CTGAAGTGTTTGGGGGAACTC | ath-miR395a | 35.8 | 23.0 |
| SL2.40ch02 | 27533088 | 27533108 | - | CTGAAGTGTTTGGGGGAACTC | ath-miR395a | 35.8 | 23.0 |
| SL2.40ch02 | 27921894 | 27921914 | - | TATTGGCCTGGTTCACTCAGA | ath-miR170-5p | 88.1 | 165.9 |
| SL2.40ch02 | 41330647 | 41330667 | - | TGATTGAGCCGTGCCAATATC | ath-miR170-3p | 49.0 | 82.9 |
| SL2.40ch02 | 41330716 | 41330736 | - | TATTGGCCTGGTTCACTCAGA | ath-miR170-5p | 88.1 | 165.9 |
| SL2.40ch02 | 46484060 | 46484083 | + | TAGTTTTTGGACGGCAGGGGCACC | stu-miR8006-5p | 37.9 | 30.0 |
| SL2.40ch02 | 46484061 | 46484083 | + | AGTTTTTGGACGGCAGGGGCACC | stu-miR8006-5p | 44.3 | 30.0 |
| SL2.40ch02 | 46484063 | 46484083 | + | TTTTTGGACGGCAGGGGCACC | stu-miR8006-5p | 44.7 | 21.0 |
| SL2.40ch03 | 292996 | 293017 | - | TCGGACCAGGCTTCATTCCCCT | ctr-miR166 | 59.2 | 99.9 |
| SL2.40ch03 | 292997 | 293017 | - | TCGGACCAGGCTTCATTCCCC | ath-miR165a-3p | 1249274.0 | 1347676.9 |
| SL2.40ch03 | 292998 | 293018 | - | CTCGGACCAGGCTTCATTCCC | ppt-miR166j | 17.5 | 44.0 |
| SL2.40ch03 | 292999 | 293019 | - | TCTCGGACCAGGCTTCATTCC | gma-miR166h-3p | 736984.2 | 1536039.9 |
| SL2.40ch03 | 6667805 | 6667825 | + | TGATTGAGCCGTGCCAATATC | ath-miR170-3p | 49.0 | 82.9 |
| SL2.40ch03 | 30608183 | 30608203 | + | CTGAAGTGTTTGGGGGAACTC | ath-miR395a | 35.8 | 23.0 |
| SL2.40ch03 | 30608715 | 30608735 | + | CTGAAGTGTTTGGGGGAACTC | ath-miR395a | 35.8 | 23.0 |
| SL2.40ch03 | 46023560 | 46023581 | - | TCGGACCAGGCTTCATTCCCCC | ctr-miR166 | 123.9 | 159.9 |
| SL2.40ch03 | 46023561 | 46023581 | - | TCGGACCAGGCTTCATTCCCC | ath-miR165a-3p | 1249274.0 | 1347676.9 |
| SL2.40ch03 | 46023562 | 46023582 | - | TTCGGACCAGGCTTCATTCCC | ppt-miR166j | 21660.3 | 25345.5 |
| SL2.40ch03 | 46023563 | 46023583 | - | CTTCGGACCAGGCTTCATTCC | gma-miR166h-3p | 358.5 | 618.5 |
| SL2.40ch03 | 49898620 | 49898640 | + | TTGGACTGAAGGGAGCTCCTT | ath-miR319a | 51.5 | 50.0 |
| SL2.40ch03 | 58491545 | 58491565 | + | GGAGGCAGCGGTTCATCGATC | ath-miR162a-5p | 74.9 | 63.9 |
| SL2.40ch03 | 58491605 | 58491625 | + | TCGATAAACCTCTGCATCCAG | ath-miR162a-3p | 2201.8 | 2051.3 |
| SL2.40ch03 | 58819366 | 58819386 | + | TTTCCAATTCCACCCATTCCT | gma-miR482a-3p | 57.9 | 48.0 |
| SL2.40ch03 | 58819366 | 58819387 | + | TTTCCAATTCCACCCATTCCTA | gma-miR482a-3p | 2521.6 | 1343.9 |
| SL2.40ch03 | 61786221 | 61786241 | + | TTTTGGATTGAAGGGAGCTCT | gma-miR319p | 50.7 | 115.9 |
| SL2.40ch03 | 61786222 | 61786242 | + | TTTGGATTGAAGGGAGCTCTA | ath-miR159a | 3506.7 | 7106.0 |
| SL2.40ch04 | 423141 | 423162 | + | TCCCCAGTCCAGGCATTCCAAC | sly-miR5300 | 2392.6 | 1580.7 |
| SL2.40ch04 | 423142 | 423162 | + | CCCCAGTCCAGGCATTCCAAC | sly-miR5300 | 294.6 | 175.9 |
| SL2.40ch04 | 2629512 | 2629533 | - | TCTTGCCTACACCGCCCATGCC | ghr-miR482b | 16564.8 | 18405.4 |
| SL2.40ch04 | 2629513 | 2629533 | - | TCTTGCCTACACCGCCCATGC | ghr-miR482b | 63.4 | 61.9 |
| SL2.40ch04 | 46803277 | 46803300 | - | TAGTTTTTGGACTGCAGGGGCACC | stu-miR8006-5p | 364.0 | 34.0 |
| SL2.40ch04 | 55142268 | 55142288 | + | TGTGGGTGGGGTGGAAAGATT | sly-miR482e-5p | 100.0 | 108.9 |
| SL2.40ch04 | 55142317 | 55142337 | + | TCTTTCCTACTCCTCCCATAC | ath-miR472-3p | 70.7 | 113.9 |
| SL2.40ch04 | 55142317 | 55142338 | + | TCTTTCCTACTCCTCCCATACC | ath-miR472-3p | 42439.0 | 48912.4 |
| SL2.40ch05 | 1703003 | 1703023 | + | CTGAAGTGTTTGGGGGAACTC | ath-miR395a | 35.8 | 23.0 |
| SL2.40ch05 | 1703180 | 1703200 | + | CTGAAGTGTTTGGGGGAACTC | ath-miR395a | 35.8 | 23.0 |
| SL2.40ch05 | 1705348 | 1705368 | + | CTGAAGTGTTTGGGGGAACTC | ath-miR395a | 35.8 | 23.0 |
| SL2.40ch05 | 1708765 | 1708785 | + | CTGAAGTGTTTGGGGGAACTC | ath-miR395a | 35.8 | 23.0 |
| SL2.40ch05 | 1713756 | 1713776 | + | CTGAAGTGTTTGGGGGAACTC | ath-miR395a | 35.8 | 23.0 |
| SL2.40ch05 | 1716672 | 1716692 | + | CTGAAGTGTTTGGGGGAACTC | ath-miR395a | 35.8 | 23.0 |
| SL2.40ch05 | 1716848 | 1716868 | + | CTGAAGTGTTTGGGGGAACTC | ath-miR395a | 35.8 | 23.0 |
| SL2.40ch05 | 2564278 | 2564298 | - | CTCATTGTCTGTTCGACCTTG | ath-miR858b | 40.0 | 36.0 |
| SL2.40ch05 | 2723775 | 2723795 | - | TTGGACTGAAGGGAGCTCCCT | ath-miR319a | 255.0 | 397.7 |
| SL2.40ch05 | 2723776 | 2723796 | - | CTTGGACTGAAGGGAGCTCCC | sof-miR159c | 524.5 | 1057.1 |
| SL2.40ch05 | 7619343 | 7619363 | - | TTTTTGGACGGCAGGGGCACC | stu-miR8006-5p | 44.7 | 21.0 |
| SL2.40ch05 | 7619343 | 7619365 | - | AGTTTTTGGACGGCAGGGGCACC | stu-miR8006-5p | 44.3 | 30.0 |
| SL2.40ch05 | 7619343 | 7619366 | - | TAGTTTTTGGACGGCAGGGGCACC | stu-miR8006-5p | 37.9 | 30.0 |
| SL2.40ch05 | 26281550 | 26281573 | + | TAGTTTTTGGACGGCAGGGGCACC | stu-miR8006-5p | 37.9 | 30.0 |
| SL2.40ch05 | 26281551 | 26281573 | + | AGTTTTTGGACGGCAGGGGCACC | stu-miR8006-5p | 44.3 | 30.0 |
| SL2.40ch05 | 26281553 | 26281573 | + | TTTTTGGACGGCAGGGGCACC | stu-miR8006-5p | 44.7 | 21.0 |
| SL2.40ch05 | 62171440 | 62171460 | - | ATCATGCTATCCCTTTGGACT | ath-miR393a-3p | 192.9 | 39.0 |
| SL2.40ch05 | 64582753 | 64582773 | - | TGCCTGGCTCCCTGTATGCCA | ath-miR160a-5p | 79.6 | 164.9 |
| SL2.40ch06 | 1372252 | 1372272 | - | AAGCTCAGGAGGGATAGCGCC | ath-miR390a-5p | 255.4 | 667.4 |
| SL2.40ch06 | 1551841 | 1551861 | + | TGTGAATGATGCGGGAGATAA | mtr-miR4414b | 33.2 | 33.0 |
| SL2.40ch06 | 1551883 | 1551903 | + | CTTGGACTGAAGGGAGCTCCC | sof-miR159c | 524.5 | 1057.1 |
| SL2.40ch06 | 1551884 | 1551904 | + | TTGGACTGAAGGGAGCTCCCT | ath-miR319a | 255.0 | 397.7 |
| SL2.40ch06 | 1551884 | 1551905 | + | TTGGACTGAAGGGAGCTCCCTT | gma-miR319g | 52.4 | 61.9 |
| SL2.40ch06 | 2516288 | 2516309 | - | TCGGACCAGGCTTCATTCCCCT | ctr-miR166 | 59.2 | 99.9 |
| SL2.40ch06 | 2516289 | 2516309 | - | TCGGACCAGGCTTCATTCCCC | ath-miR165a-3p | 1249274.0 | 1347676.9 |
| SL2.40ch06 | 2516290 | 2516310 | - | TTCGGACCAGGCTTCATTCCC | ppt-miR166j | 21660.3 | 25345.5 |
| SL2.40ch06 | 2516290 | 2516311 | - | TTTCGGACCAGGCTTCATTCCC | ata-miR166b-5p | 6038.0 | 6663.3 |
| SL2.40ch06 | 2516291 | 2516311 | - | TTTCGGACCAGGCTTCATTCC | gma-miR166h-3p | 1619.0 | 9776.7 |
| SL2.40ch06 | 23942469 | 23942489 | - | TTTTTGGACGGCAGGGGCACC | stu-miR8006-5p | 44.7 | 21.0 |
| SL2.40ch06 | 23942469 | 23942491 | - | AGTTTTTGGACGGCAGGGGCACC | stu-miR8006-5p | 44.3 | 30.0 |
| SL2.40ch06 | 23942469 | 23942492 | - | TAGTTTTTGGACGGCAGGGGCACC | stu-miR8006-5p | 37.9 | 30.0 |
| SL2.40ch06 | 33130361 | 33130381 | + | TTTCGGACCAGGCTTCATTCC | gma-miR166h-3p | 1619.0 | 9776.7 |
| SL2.40ch06 | 33130361 | 33130382 | + | TTTCGGACCAGGCTTCATTCCC | ata-miR166b-5p | 6038.0 | 6663.3 |
| SL2.40ch06 | 33130362 | 33130382 | + | TTCGGACCAGGCTTCATTCCC | ppt-miR166j | 21660.3 | 25345.5 |
| SL2.40ch06 | 33130363 | 33130383 | + | TCGGACCAGGCTTCATTCCCC | ath-miR165a-3p | 1249274.0 | 1347676.9 |
| SL2.40ch06 | 33130363 | 33130384 | + | TCGGACCAGGCTTCATTCCCCC | ctr-miR166 | 123.9 | 159.9 |
| SL2.40ch06 | 33869922 | 33869943 | - | TTTCCTATTCCACCCATGCCAA | stu-miR482c | 374.2 | 375.7 |
| SL2.40ch06 | 33870237 | 33870258 | - | TCTTGCCTACACCGCCCATGCC | ghr-miR482b | 16564.8 | 18405.4 |
| SL2.40ch06 | 33870238 | 33870258 | - | TCTTGCCTACACCGCCCATGC | ghr-miR482b | 63.4 | 61.9 |
| SL2.40ch06 | 33877995 | 33878016 | - | TCTTGCCAATACCGCCCATTCC | gma-miR482a-3p | 1195.9 | 935.2 |
| SL2.40ch06 | 39152013 | 39152033 | + | TGATTGAGCCGTGCCAATATC | ath-miR170-3p | 49.0 | 82.9 |
| SL2.40ch06 | 39463120 | 39463140 | + | GGAGGCAGCGGTTCATCGATC | ath-miR162a-5p | 74.9 | 63.9 |
| SL2.40ch06 | 39463195 | 39463215 | + | TCGATAAACCTCTGCATCCAG | ath-miR162a-3p | 2201.8 | 2051.3 |
| SL2.40ch06 | 40810831 | 40810851 | + | TGATTGAGCCGTGCCAATATC | ath-miR170-3p | 49.0 | 82.9 |
| SL2.40ch06 | 43022443 | 43022463 | - | TGCCTGGCTCCCTGTATGCCT | ath-miR160a-5p | 37.0 | 92.9 |
| SL2.40ch06 | 45629433 | 45629453 | - | TGAAGCTGCCAGCATGATCTA | ath-miR167a-5p | 272.5 | 96.9 |
| SL2.40ch07 | 235722 | 235742 | - | AGGCTGTGATGATGATGATGA | atr-miR8577 | 86.8 | 70.9 |
| SL2.40ch07 | 2628853 | 2628873 | - | TTCCACAGCTTTCTTGAACTT | ath-miR396a-5p | 366.5 | 242.8 |
| SL2.40ch07 | 56157226 | 56157246 | - | TTTTTGGACGGCAGGGGCACC | stu-miR8006-5p | 44.7 | 21.0 |
| SL2.40ch07 | 56157226 | 56157248 | - | AGTTTTTGGACGGCAGGGGCACC | stu-miR8006-5p | 44.3 | 30.0 |
| SL2.40ch07 | 56157226 | 56157249 | - | TAGTTTTTGGACGGCAGGGGCACC | stu-miR8006-5p | 37.9 | 30.0 |
| SL2.40ch07 | 58137146 | 58137166 | + | TGATTGAGCCGTGCCAATATC | ath-miR170-3p | 49.0 | 82.9 |
| SL2.40ch08 | 558760 | 558780 | + | TCGCTTGGTGCAGGTCGGGAC | ath-miR168a-5p | 2868.9 | 2747.7 |
| SL2.40ch08 | 2978490 | 2978510 | + | CGTCGGACCAGGCTTCATTCC | gma-miR166h-3p | 52.8 | 112.9 |
| SL2.40ch08 | 2978491 | 2978511 | + | GTCGGACCAGGCTTCATTCCC | ppt-miR166j | 25.5 | 44.0 |
| SL2.40ch08 | 2978492 | 2978512 | + | TCGGACCAGGCTTCATTCCCC | ath-miR165a-3p | 1249274.0 | 1347676.9 |
| SL2.40ch08 | 2978492 | 2978513 | + | TCGGACCAGGCTTCATTCCCCT | ctr-miR166 | 59.2 | 99.9 |
| SL2.40ch08 | 46920291 | 46920311 | - | TTTTTGGACGGCAGGGGCACC | stu-miR8006-5p | 44.7 | 21.0 |
| SL2.40ch08 | 46920291 | 46920313 | - | AGTTTTTGGACGGCAGGGGCACC | stu-miR8006-5p | 44.3 | 30.0 |
| SL2.40ch08 | 46920291 | 46920314 | - | TAGTTTTTGGACGGCAGGGGCACC | stu-miR8006-5p | 37.9 | 30.0 |
| SL2.40ch08 | 46958924 | 46958944 | - | TTTTTGGACGGCAGGGGCACC | stu-miR8006-5p | 44.7 | 21.0 |
| SL2.40ch08 | 46958924 | 46958946 | - | AGTTTTTGGACGGCAGGGGCACC | stu-miR8006-5p | 44.3 | 30.0 |
| SL2.40ch08 | 46958924 | 46958947 | - | TAGTTTTTGGACGGCAGGGGCACC | stu-miR8006-5p | 37.9 | 30.0 |
| SL2.40ch08 | 53776411 | 53776431 | + | TGTCGCAGATGACTTTCGCCC | sly-miR1919c-5p | 538.5 | 494.6 |
| SL2.40ch08 | 53776477 | 53776497 | + | ACGAGAGTCATCTGTGACAGG | sly-miR1919a | 22.1 | 23.0 |
| SL2.40ch08 | 53789929 | 53789949 | + | TGTCGCAGATGACTTTCGCCC | sly-miR1919c-5p | 538.5 | 494.6 |
| SL2.40ch08 | 53789995 | 53790015 | + | ACGAGAGTCATCTGTGACAGG | sly-miR1919a | 22.1 | 23.0 |
| SL2.40ch08 | 53821575 | 53821595 | + | ACGAGAGTCATCTGTGACAGG | sly-miR1919a | 22.1 | 23.0 |
| SL2.40ch08 | 59305571 | 59305592 | - | TTTTGTTCGCAGATACTACAGT | sly-miR9474-3p | 30.7 | 23.0 |
| SL2.40ch08 | 61949534 | 61949554 | - | TTGGACTGAAGGGAGCTCCCT | ath-miR319a | 255.0 | 397.7 |
| SL2.40ch08 | 61949535 | 61949555 | - | CTTGGACTGAAGGGAGCTCCC | sof-miR159c | 524.5 | 1057.1 |
| SL2.40ch08 | 62860631 | 62860651 | + | GGAATGTTGTCTGGCTCGAGG | ath-miR165a-5p | 525.8 | 431.6 |
| SL2.40ch08 | 62860744 | 62860764 | + | TGTCGGACCAGGCTTCATTCC | gma-miR166h-3p | 1622.9 | 12018.8 |
| SL2.40ch08 | 62860745 | 62860765 | + | GTCGGACCAGGCTTCATTCCC | ppt-miR166j | 25.5 | 44.0 |
| SL2.40ch08 | 62860746 | 62860766 | + | TCGGACCAGGCTTCATTCCCC | ath-miR165a-3p | 1249274.0 | 1347676.9 |
| SL2.40ch08 | 62860746 | 62860767 | + | TCGGACCAGGCTTCATTCCCCC | ctr-miR166 | 123.9 | 159.9 |
| SL2.40ch09 | 3988514 | 3988534 | + | TTTTTGGACGGCAGGGGCACC | stu-miR8006-5p | 44.7 | 21.0 |
| SL2.40ch09 | 4192014 | 4192037 | + | TAGTTTTTGGACGGCAGGGGCACC | stu-miR8006-5p | 37.9 | 30.0 |
| SL2.40ch09 | 4192015 | 4192037 | + | AGTTTTTGGACGGCAGGGGCACC | stu-miR8006-5p | 44.3 | 30.0 |
| SL2.40ch09 | 4192017 | 4192037 | + | TTTTTGGACGGCAGGGGCACC | stu-miR8006-5p | 44.7 | 21.0 |
| SL2.40ch09 | 7197977 | 7197997 | + | TGGAGAAGCAGGGCACGTGCA | ath-miR164a | 1233.7 | 465.6 |
| SL2.40ch09 | 7198118 | 7198138 | + | CATGTGCCTGTTTTCCCCATC | sly-miR164a-3p | 200.5 | 55.0 |
| SL2.40ch09 | 8388200 | 8388220 | + | AAGCTCAGGAGGGATAGCGCC | ath-miR390a-5p | 255.4 | 667.4 |
| SL2.40ch09 | 35878620 | 35878641 | + | TGGGGGCTCGAAGACGATCAGA | peu-miR2916 | 51.9 | 37.0 |
| SL2.40ch09 | 35878621 | 35878641 | + | GGGGGCTCGAAGACGATCAGA | peu-miR2916 | 30.7 | 34.0 |
| SL2.40ch09 | 59575910 | 59575930 | + | TGAAGCTGCCAGCATGATCTA | ath-miR167a-5p | 272.5 | 96.9 |
| SL2.40ch09 | 59584688 | 59584708 | + | TGAAGCTGCCAGCATGATCTA | ath-miR167a-5p | 272.5 | 96.9 |
| SL2.40ch09 | 63883415 | 63883435 | + | TGAAGCTGCCAGCATGATCTA | ath-miR167a-5p | 272.5 | 96.9 |
| SL2.40ch09 | 64446633 | 64446654 | - | TCGGACCAGGCTTCATTCCCCT | ctr-miR166 | 59.2 | 99.9 |
| SL2.40ch09 | 64446634 | 64446654 | - | TCGGACCAGGCTTCATTCCCC | ath-miR165a-3p | 1249274.0 | 1347676.9 |
| SL2.40ch09 | 64446635 | 64446655 | - | TTCGGACCAGGCTTCATTCCC | ppt-miR166j | 21660.3 | 25345.5 |
| SL2.40ch09 | 64446635 | 64446656 | - | TTTCGGACCAGGCTTCATTCCC | ata-miR166b-5p | 6038.0 | 6663.3 |
| SL2.40ch09 | 64446636 | 64446656 | - | TTTCGGACCAGGCTTCATTCC | gma-miR166h-3p | 1619.0 | 9776.7 |
| SL2.40ch10 | 3321758 | 3321778 | + | GGAATGTTGTCTGGCTCGAGG | ath-miR165a-5p | 525.8 | 431.6 |
| SL2.40ch10 | 3321907 | 3321927 | + | CGTCGGACCAGGCTTCATTCC | gma-miR166h-3p | 52.8 | 112.9 |
| SL2.40ch10 | 3321908 | 3321928 | + | GTCGGACCAGGCTTCATTCCC | ppt-miR166j | 25.5 | 44.0 |
| SL2.40ch10 | 3321909 | 3321929 | + | TCGGACCAGGCTTCATTCCCC | ath-miR165a-3p | 1249274.0 | 1347676.9 |
| SL2.40ch10 | 3321909 | 3321930 | + | TCGGACCAGGCTTCATTCCCCT | ctr-miR166 | 59.2 | 99.9 |
| SL2.40ch10 | 61922244 | 61922264 | + | AATGAAGACTGATCCAAGATC | osa-miR166g-5p | 17.9 | 22.0 |
| SL2.40ch10 | 61922622 | 61922642 | + | TCTCGGACCAGGCTTCATTCC | gma-miR166h-3p | 736984.2 | 1536039.9 |
| SL2.40ch10 | 61922624 | 61922644 | + | TCGGACCAGGCTTCATTCCTC | ath-miR165a-3p | 35012.7 | 46459.5 |
| SL2.40ch10 | 62182533 | 62182554 | + | ATGGGTAGCACAAGGATTAATG | sly-miR6027-5p | 518.1 | 412.6 |
| SL2.40ch10 | 62182634 | 62182654 | + | TGAATCCTTCGGCTATCCATA | sly-miR6027-3p | 102.2 | 86.9 |
| SL2.40ch10 | 62182634 | 62182655 | + | TGAATCCTTCGGCTATCCATAA | sly-miR6027-3p | 112.8 | 91.9 |
| SL2.40ch11 | 17806132 | 17806153 | + | TGGGGGCTCGAAGACGATCAGA | peu-miR2916 | 51.9 | 37.0 |
| SL2.40ch11 | 17806133 | 17806153 | + | GGGGGCTCGAAGACGATCAGA | peu-miR2916 | 30.7 | 34.0 |
| SL2.40ch11 | 17806132 | 17806154 | + | TGGGGGCTCGAAGACGATCAGAT | peu-miR2916 | 60.5 | 31.0 |
| SL2.40ch11 | 17806133 | 17806154 | + | GGGGGCTCGAAGACGATCAGAT | peu-miR2916 | 40.9 | 39.0 |
| SL2.40ch11 | 17806134 | 17806154 | + | GGGGCTCGAAGACGATCAGAT | peu-miR2916 | 15.8 | 22.0 |
| SL2.40ch11 | 47382919 | 47382939 | - | TATGTTCTCAGGTCGCCCCTG | ath-miR398b-3p | 50.7 | 70.9 |
| SL2.40ch11 | 50724819 | 50724839 | - | TGCATTTGCACCTGCACCTCC | csi-miR530a | 123.5 | 50.0 |
| SL2.40ch11 | 51189470 | 51189490 | + | TGCCTGGCTCCCTGTATGCCT | ath-miR160a-5p | 37.0 | 92.9 |
| SL2.40ch12 | 519799 | 519819 | - | TTGAGCCGCGCCAATATCACG | ath-miR171b-3p | 18.7 | 36.0 |
| SL2.40ch12 | 1977757 | 1977777 | - | TTGGCTGAGTGAGCATCACGG | sly-miR9471b-3p | 66.8 | 63.9 |
| SL2.40ch12 | 2899110 | 2899130 | + | TTCCACAGCTTTCTTGAACTT | ath-miR396a-5p | 366.5 | 242.8 |
| SL2.40ch12 | 2905770 | 2905790 | - | TTCCACAGCTTTCTTGAACTG | ath-miR396a-5p | 1170.7 | 421.6 |
| SL2.40ch12 | 3071186 | 3071209 | + | TAGTTTTTGGACGGCAGGGGCACC | stu-miR8006-5p | 37.9 | 30.0 |
| SL2.40ch12 | 3071187 | 3071209 | + | AGTTTTTGGACGGCAGGGGCACC | stu-miR8006-5p | 44.3 | 30.0 |
| SL2.40ch12 | 3071189 | 3071209 | + | TTTTTGGACGGCAGGGGCACC | stu-miR8006-5p | 44.7 | 21.0 |
| SL2.40ch12 | 6988872 | 6988892 | - | ACGAGAGTCATCTGTGACAGG | sly-miR1919a | 22.1 | 23.0 |
| SL2.40ch12 | 6988948 | 6988968 | - | TGTCGCAGATGACTTTCGCCC | sly-miR1919c-5p | 538.5 | 494.6 |
| SL2.40ch12 | 47456665 | 47456685 | - | TTGGACTGAAGGGAGCTCCCT | ath-miR319a | 255.0 | 397.7 |
| SL2.40ch12 | 47456666 | 47456686 | - | CTTGGACTGAAGGGAGCTCCC | sof-miR159c | 524.5 | 1057.1 |
| SL2.40ch12 | 62926475 | 62926495 | + | TTTTTGGACGGCAGGGGCACC | stu-miR8006-5p | 44.7 | 21.0 |
| SL2.40ch12 | 63569025 | 63569045 | + | TCGCTTGGTGCAGGTCGGGAC | ath-miR168a-5p | 2868.9 | 2747.7 |
| SL2.40ch12 | 63569139 | 63569160 | + | TCCCGCCTTGCATCAACTGAAT | osa-miR168a-3p | 57.0 | 44.0 |
| SL2.40ch12 | 63569140 | 63569160 | + | CCCGCCTTGCATCAACTGAAT | ath-miR168a-3p | 157.5 | 45.0 |

**Table S2**. List of the new miRNAs identified from WT anthers.

| **Locus** | **Start** | **Stop** | **miRNA sequence** | **Normalized Expression** | | **DE**** | **Designation** |
| --- | --- | --- | --- | --- | --- | --- | --- |
|  |  |  |  | **WT** | ***7B-1*** |  |  |
| SL2.40ch02 | 49362089 | 49362109 | TTCTCCAGCCATTGTTTGTTT | 34.4 | 0.8 | 1.4 | Mir#W |
| SL2.40ch08 | 55732501 | 55732521 | TTTCTACCCCTTTTCGGCCTT | 11.7 | 1.7 | 0.5 |  |
| SL2.40ch01 | 14863601 | 14863621 | TATGCCTCTGACTTTCCTTCT | 14.5 | 4.2 | 0.5 |  |
| SL2.40ch11 | 7833133 | 7833154 | TTCTAGAATTCTCCACAAGCCT | 11.7 | 3.3 | 0.4 |  |
| SL2.40ch05 | 62780295 | 62780316 | TCCGACTGGCAAAGGACTTAGC | 19.5 | 9.2 | 0.4 |  |
| SL2.40ch10 | 4823991 | 4824012 | AGTACGGTAGGGGCAGAGGGAA | 30.9 | 18.3 | 0.4 |  |
| SL2.40ch04 | 94350 | 94371 | TTACCCCCGAACCCATTTTTTT | 12.1 | 4.2 | 0.4 |  |
| SL2.40ch03 | 61794716 | 61794736 | TTGGACTGAAGGGTTTCCTTC | 23.4 | 15.0 | 0.3 |  |
| SL2.40ch12 | 16028421 | 16028442 | ATCGGCGCCTGACCCTGAGATG | 19.2 | 12.5 | 0.3 |  |
| SL2.40ch10 | 1709809 | 1709830 | TAACTTCGTCTAGCTCGCCTTC | 870.6 | 790.2 | 0.1 | miR#A |
|  |  |  | AGGGGAGATAGATGAAGTTAGG*(3) |  |  |  |  |
| SL2.40ch01 | 66175618 | 66175639 | CACCGATGAGGATTTGGCCCCG | 22.4 | 19.2 | 0.1 |  |
| SL2.40ch11 | 31406729 | 31406749 | CCCTCGTGTTTAGTTGCCATC | 130.2 | 127.4 | 0.0 |  |
| SL2.40ch08 | 55643345 | 55643365 | TGGGACAAGGCGCCTCGAGGT | 2.5 | 3.3 | -0.1 |  |
| SL2.40ch08 | 60344195 | 60344216 | TGTTTGAGCTGTAGGAAGAAGG | 8.9 | 10.8 | -0.1 |  |
| SL2.40ch07 | 21405514 | 21405535 | GTAGCGAAATTCCTTGTCGGGT | 5.7 | 7.5 | -0.1 |  |
| SL2.40ch04 | 54853451 | 54853472 | TCTTCATCTGAAGCGAGCGCCT | 29.1 | 43.3 | -0.4 |  |
| SL2.40ch10 | 35829161 | 35829182 | AACCATTGGGACTGTAACTCGA | 2.5 | 10.0 | -0.4 |  |
| SL2.40ch04 | 3142591 | 3142611 | TGGGGAGGGTGGTGTGTACGC | 7.1 | 17.5 | -0.5 | miR#B |
|  |  |  | GTACATCCTACCCTCCCCAGA*(6) |  |  |  |  |

Highlighted miRNAs were present in both WT and *7B-1* libraries.

* Indicates miRNA* and numbers in the parenthesis are the raw read counts.

** DE is differential expression values which were calculated as log2-fold changes of the expression. Negative and positive values mean up- and down regulation of the expression in *7B-1*, respectively. DE value of ±1 was considered as a cutoff value for significant changes of the expression.

**Table S3**. List of the new miRNAs identified from *7B-1* anthers.

| **Locus** | **Start** | **Stop** | **miRNA sequence** | **Normalized Expression** | | | **DE**** | **Designation** |
| --- | --- | --- | --- | --- | --- | --- | --- | --- |
|  |  |  |  | **WT** | ***7B-1*** | |  |  |
| SL2.40ch06 | 32482742 | 32482763 | TCCGACTGAGAATTGCAAAGGT | 226.0 | 15.8 | 2.78 | | mir#M |
| SL2.40ch02 | 20444357 | 20444378 | TGAATGATGAGGAGATGAACTT | 22.4 | 7.5 | 0.62 | |  |
| SL2.40ch03 | 61794716 | 61794736 | TTGGACTGAAGGGTTTCCTTC | 23.4 | 15.0 | 0.31 | |  |
| SL2.40ch11 | 13374075 | 13374094 | TTGCGTCGTTGTGCCTGGGC | 41.5 | 34.1 | 0.18 | |  |
| SL2.40ch10 | 1709809 | 1709830 | TAACTTCGTCTAGCTCGCCTTC | 870.6 | 790.2 | 0.14 | | mir#A |
|  |  |  | AGGGGAGATAGATGAAGTTAGG*(3) |  |  |  | |  |
| SL2.40ch01 | 66175618 | 66175639 | CACCGATGAGGATTTGGCCCCG | 22.4 | 19.2 | 0.11 | |  |
| SL2.40ch08 | 60344195 | 60344216 | TGTTTGAGCTGTAGGAAGAAGG | 8.9 | 10.8 | -0.09 | |  |
| SL2.40ch04 | 54853451 | 54853472 | TCTTCATCTGAAGCGAGCGCCT | 29.1 | 43.3 | -0.37 | |  |
| SL2.40ch10 | 35829161 | 35829182 | AACCATTGGGACTGTAACTCGA | 2.5 | 10.0 | -0.42 | |  |
| SL2.40ch12 | 62673607 | 62673627 | CTCTGTAGAGCTTGGTGCAAT | 1.8 | 10.0 | -0.46 | |  |
| SL2.40ch04 | 3142591 | 3142611 | TGGGGAGGGTGGTGTGTACGC | 7.1 | 17.5 | -0.47 | | mir#B |
|  |  |  | TATGCGTACATCCTACCCTCC*(2) |  |  |  | |  |
|  |  |  | TGCGTACATCCTACCCTCCCC*(2) |  |  |  | |  |
| SL2.40ch01 | 58340333 | 58340354 | AGTGCATCCCGATTGTTCTCTC | 3.5 | 26.6 | -0.99 | |  |

Highlighted miRNAs were present in both WT and *7B-1* libraries.

* indicates miRNA* and numbers in the parenthesis are the raw read counts.

**DE is differential expression values which were calculated as log2-fold changes of the expression. Negative and positive values mean up- and down regulation of the expression in *7B-1*, respectively. DE value of ±1 was considered as a cutoff value for significant changes of the expression.

**Table S4**. List of miRNA-target cleavage sites

| **miRNA** | **Target gene** | |
| --- | --- | --- |
|  | **SGN accession no** | **Cleavage site*** |
| miR166 | Solyc02g024070.2.1 | 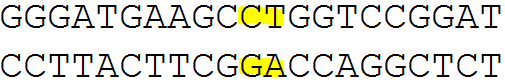 |
|  | Solyc03g006970.1.1 | 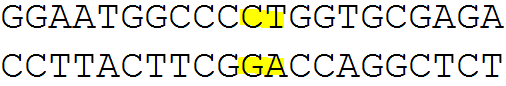 |
|  | Solyc03g116850.2.1 | 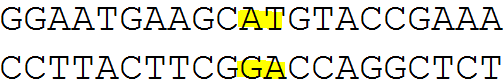 |
|  | Solyc03g025740.2.1 | 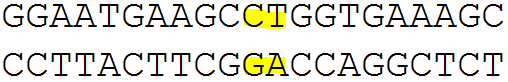 |
|  | Solyc07g045410.1.1 | 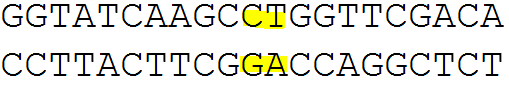 |
| miR390 | Solyc00g009090.2.1 | 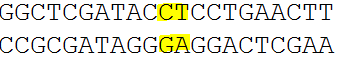 |
|  | Solyc09g091850.2.1 | 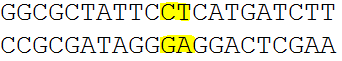 |
|  | Solyc11g016930.1.1 | 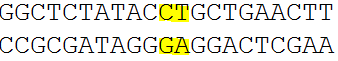 |
| miR159 | Solyc01g009070.2.1 | 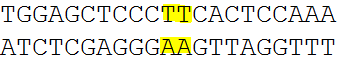 |
|  | Solyc01g102510.2.1 | 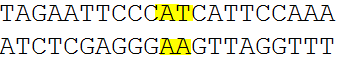 |
|  | Solyc02g078670.2.1 | 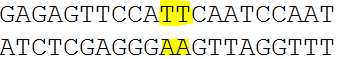 |
|  | Solyc02g090160.2.1 | 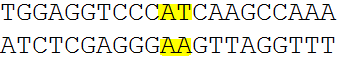 |
|  | Solyc03g043890.2.1 | 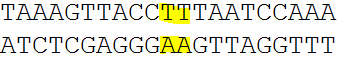 |
|  | Solyc06g073640.2.1 | 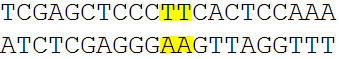 |
|  | Solyc07g052640.2.1 | 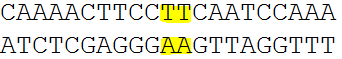 |
|  | Solyc09g082890.1.1 | 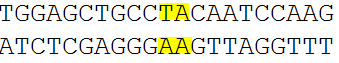 |
|  | Solyc10g019260.1.1 | 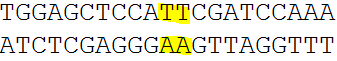 |
|  | Solyc11g072060.1.1 | 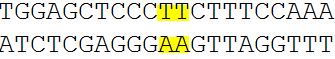 |
| miR530 | Solyc01g091550.2.1 | 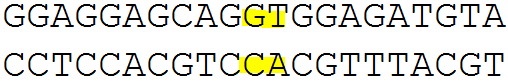 |
|  | Solyc02g083460.2.1 | 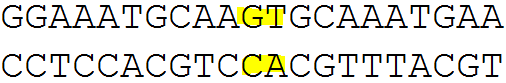 |
|  | Solyc02g084520.2.1 | 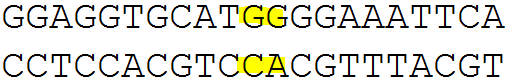 |
|  | Solyc02g085520.2.1 | 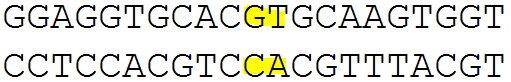 |
|  | Solyc02g086930.2.1 | 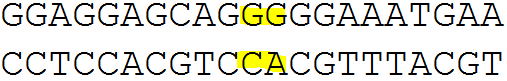 |
|  | Solyc03g019920.1.1 | 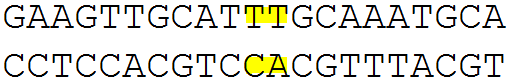 |
|  | Solyc03g093230.2.1 | 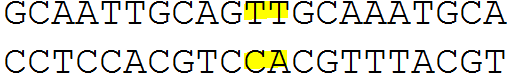 |
|  | Solyc04g009450.1.1 | 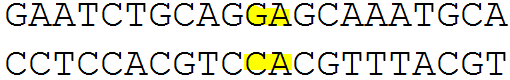 |
|  | Solyc04g081070.2.1 | 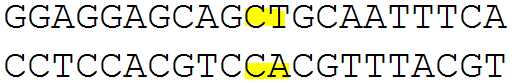 |
|  | Solyc06g063170.2.1 | 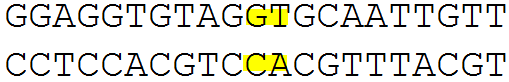 |
|  | Solyc07g006030.2.1 | 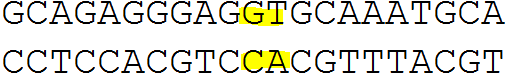 |
|  | Solyc08g007500.2.1 | 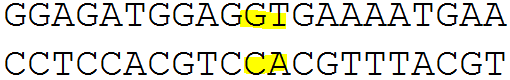 |
|  | Solyc11g062220.1.1 | 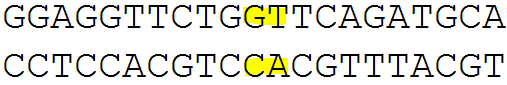 |
|  | Solyc12g008690.1.1 | 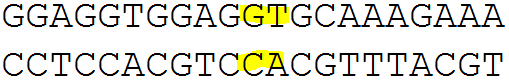 |
| miR393 | Solyc01g057310.2.1 | 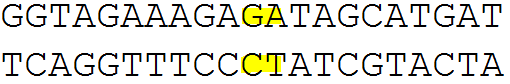 |
|  | Solyc02g088800.1.1 | 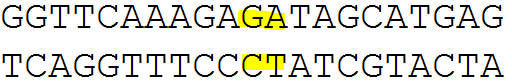 |
|  | Solyc06g068840.2.1 | 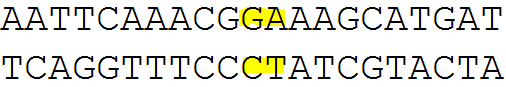 |
|  | Solyc08g081890.2.1 | 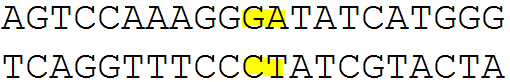 |
|  | Solyc11g006310.1.1 | 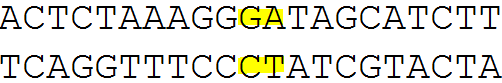 |
| miR396 | Solyc01g066500.1.1 | 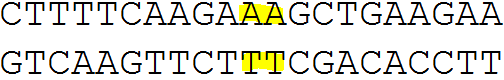 |
|  | Solyc00g105750.1.1 | 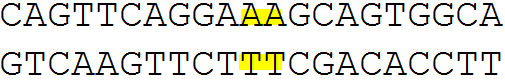 |
|  | Solyc00g071180.2.1 | 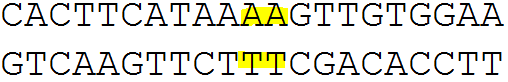 |
|  | Solyc01g090270.2.1 | 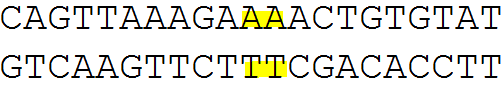 |
|  | Solyc01g094930.2.1 | 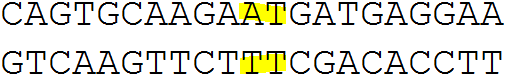 |
|  | Solyc01g110450.2.1 | 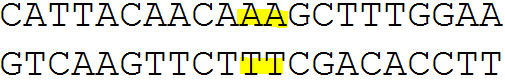 |
|  | Solyc02g023950.2.1 | 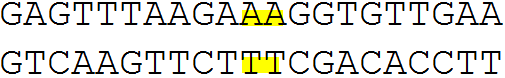 |
|  | Solyc02g083190.1.1 | 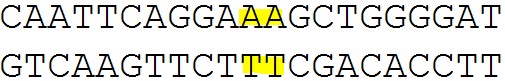 |
|  | Solyc03g058930.2.1 | 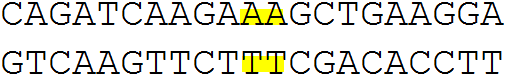 |
|  | Solyc03g114150.2.1 | 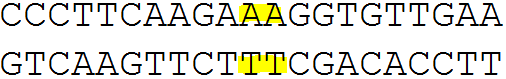 |
|  | Solyc03g118350.2.1 | 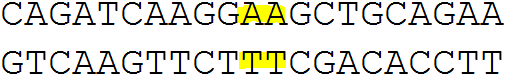 |
|  | Solyc05g053090.1.1 | 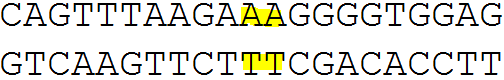 |
|  | Solyc06g007320.2.1 | 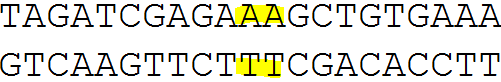 |
|  | Solyc06g059760.2.1 | 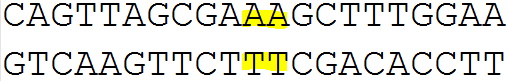 |
|  | Solyc07g019640.1.1 | 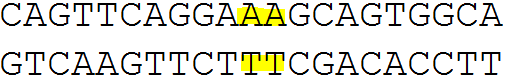 |
|  | Solyc07g045480.2.1 | 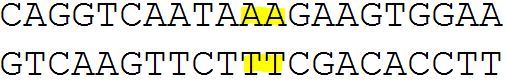 |
|  | Solyc09g057910.2.1 | 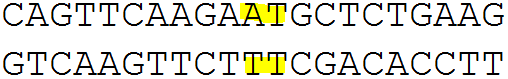 |
|  | Solyc10g047270.1.1 | 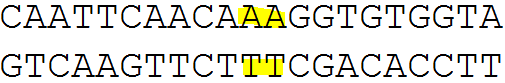 |
|  | Solyc11g006680.1.1 | 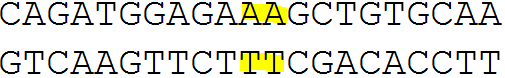 |
|  | Solyc11g020100.1.1 | 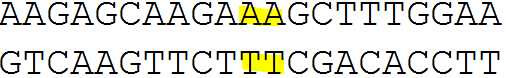 |
|  | Solyc12g013840.1.1 | 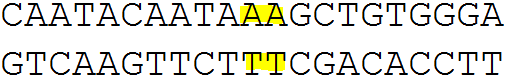 |
| miR164 | Solyc03g115850.2.1 | 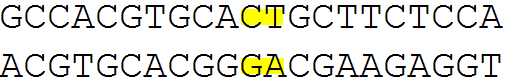 |
|  | Solyc06g084350.2.1 | 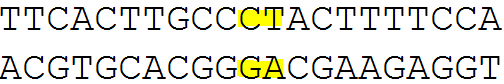 |
|  | Solyc11g066150.1.1 | 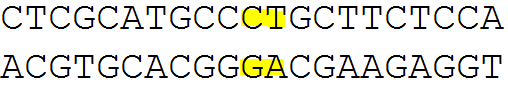 |
| miR167 | Solyc01g010020.2.1 | 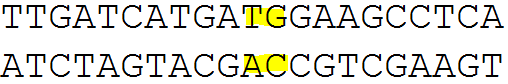 |
|  | Solyc01g086900.2.1 | 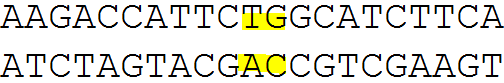 |
|  | Solyc02g037530.2.1 | 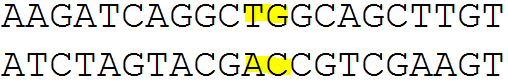 |
|  | Solyc03g007790.2.1 | 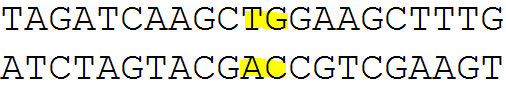 |
|  | Solyc03g117700.1.1 | 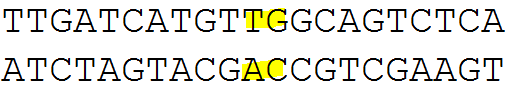 |
|  | Solyc08g065360.2.1 | 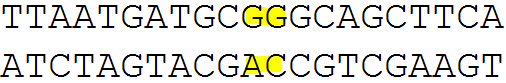 |
|  | Solyc08g069010.2.1 | 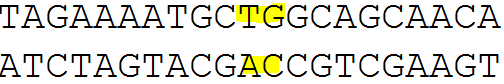 |
| miR168 | Solyc01g108250.2.1 | 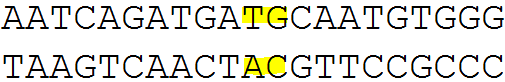 |
|  | Solyc04g078130.2.1 | 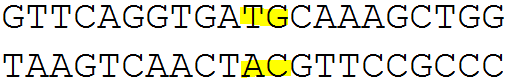 |
|  | Solyc06g053710.2.1 | 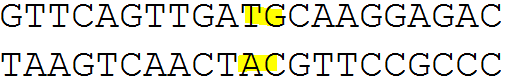 |
|  | Solyc06g060160.1.1 | 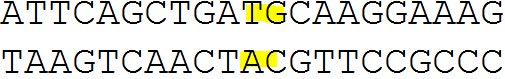 |
|  | Solyc09g018560.1.1 | 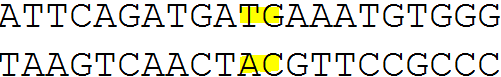 |
|  | Solyc09g090650.2.1 | 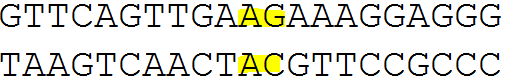 |
| miR8006 | Solyc01g008530.2.1 | 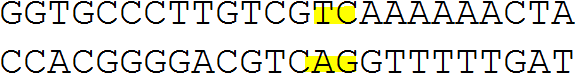 |
|  | Solyc01g008790.2.1 | 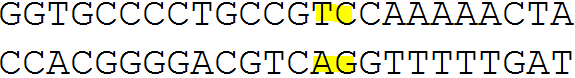 |
|  | Solyc04g078250.2.1 | 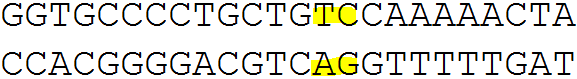 |
|  | Solyc05g014050.2.1 | 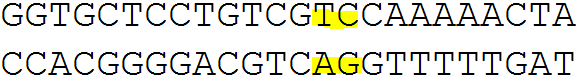 |
|  | Solyc07g049440.2.1 | 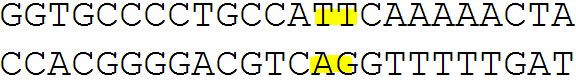 |
|  | Solyc08g078650.2.1 | 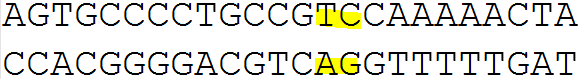 |
|  | Solyc09g064850.2.1 | 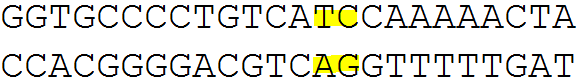 |
|  | Solyc10g009520.2.1 | 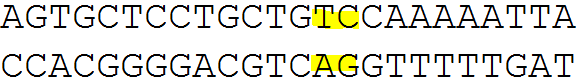 |
|  | Solyc12g013850.1.1 | 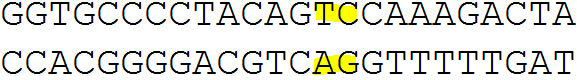 |
|  | Solyc12g088130.1.1 | 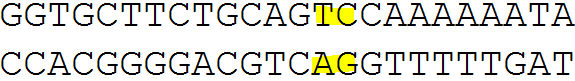 |
|  | Solyc12g096640.1.1 | 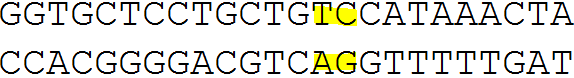 |
| miR#W | Solyc01g008240.2.1 | 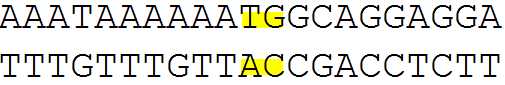 |
|  | Solyc01g015110.1.1 | 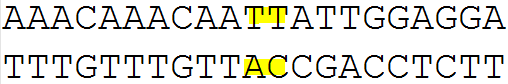 |
|  | Solyc01g079390.2.1 | 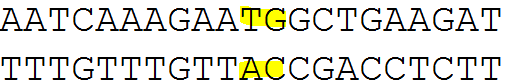 |
|  | Solyc01g095910.1.1 | 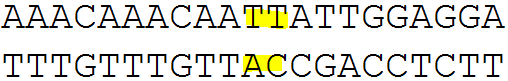 |
|  | Solyc01g103670.2.1 | 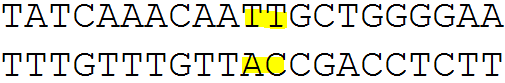 |
|  | Solyc01g103800.2.1 | 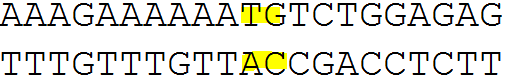 |
|  | Solyc01g104990.2.1 | 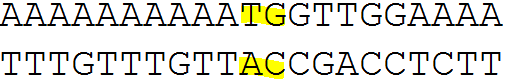 |
|  | Solyc02g022870.2.1 | 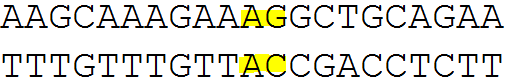 |
|  | Solyc02g069410.2.1 | 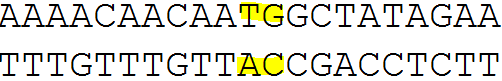 |
|  | Solyc02g069740.2.1 | 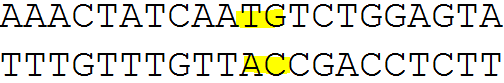 |
|  | Solyc02g092780.1.1 | 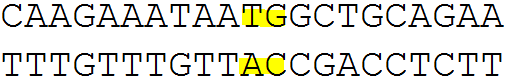 |
|  | Solyc03g078240.1.1 | 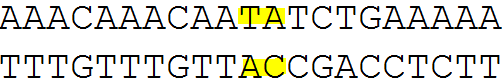 |
|  | Solyc03g115860.2.1 | 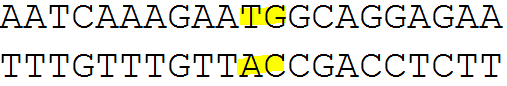 |
|  | Solyc04g074660.1.1 | 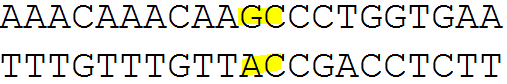 |
|  | Solyc04g079240.2.1 | 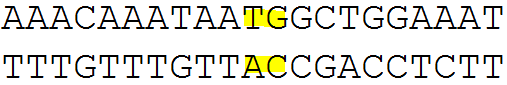 |
|  | Solyc05g007850.1.1 |  |
|  | Solyc06g016750.2.1 |  |
|  | Solyc06g065180.2.1 |  |
|  | Solyc06g083070.2.1 |  |
|  | Solyc08g014530.1.1 |  |
|  | Solyc08g069000.2.1 |  |
|  | Solyc08g081290.2.1 |  |
|  | Solyc09g007370.2.1 |  |
|  | Solyc09g083200.2.1 |  |
|  | Solyc09g089550.2.1 |  |
|  | Solyc10g084410.1.1 |  |
|  | Solyc12g027580.1.1 |  |
|  | Solyc12g077660.1.1 |  |
| miR#M | Solyc03g112990.1.1 |  |
| miR#A | Solyc03g119580.1.1 |  |
|  | Solyc10g078230.1.1 |  |
| miR#B | Solyc01g044350.2.1 |  |
|  | Solyc02g089170.2.1 |  |
|  | Solyc03g113250.2.1 |  |
|  | Solyc04g007260.2.1 |  |
|  | Solyc04g009620.2.1 |  |
|  | Solyc04g071160.2.1 |  |
|  | Solyc05g054890.2.1 |  |
|  | Solyc06g084100.2.1 |  |
|  | Solyc07g005390.2.1 |  |
|  | Solyc07g007230.2.1 |  |
|  | Solyc07g015870.2.1 |  |
|  | Solyc08g069030.2.1 |  |
|  | Solyc09g074100.2.1 |  |
|  | Solyc09g091230.2.1 |  |

* Top strand corresponds to target transcript in 5ˊ-3ˊ direction, and bottom strand corresponds to the miRNA sequence in 3ˊ-5ˊ direction. Cleavage sites are highlighted.

**Table S5**. List of ta-siRNAs and their predicted targets.

| **Ta-siRNA sequence** | **Read counts** | | **DE*** | **Target gene** | |
| --- | --- | --- | --- | --- | --- |
|  | **WT** | ***7B-1*** |  | **Accession** | **Annotation** |
| TACCCTACCAAAGTCGACATT | 19 | 0 | - | Solyc03g117790.1.1 | Serine/threonine kinase |
|  |  |  |  | Solyc09g010210.2.1 | Glycoside hydrolase |
|  |  |  |  | Solyc10g079570.1.1 | Acyltransferase |
| TTTCGGGCATAGGTTGAGGGG | 11 | 0 | - | Solyc03g026320.2.1 | Permease protein MsbA |
|  |  |  |  | Solyc05g014510.2.1 | Ankyrin repeat domain-containing protein 44 |
|  |  |  |  | Solyc06g064940.2.1 | Phosphatidylinositol transfer protein SFH5 |
|  |  |  |  | Solyc06g076100.2.1 | Protein phosphatase 2C |
|  |  |  |  | Solyc07g006070.1.1 | NAD-dependent epimerase/dehydratase |
|  |  |  |  | Solyc07g047910.1.1 | Serine/threonine protein kinase |
|  |  |  |  | Solyc10g007080.2.1 | Multi antimicrobial extrusion protein MatE |
| ATATTCTATCCTGGTGTCGGA | 91 | 0 | - | Solyc08g075570.2.1 | Urea active transporter-like protein |
| CACGGGTACTGAGTAGATATC | 11 | 0 | - | Solyc01g057780.2.1 | Zinc finger protein |
|  |  |  |  | Solyc03g013360.1.1 | Retrotransposon gag protein |
| TTCTGAGTTCACACTGTGACT | 16 | 0 | - | Solyc01g107560.2.1 | UDP-glucosyltransferase 1 |
|  |  |  |  | Solyc02g036270.2.1 | Cc-nbs-lrr** |
|  |  |  |  | Solyc03g094100.1.1 | Nbs |
|  |  |  |  | Solyc07g044800.2.1 | Nbs-lrr |
|  |  |  |  | Solyc08g075350.1.1 | Pentatricopeptide repeat-containing protein |
|  |  |  |  | Solyc08g080780.2.1 | SKIP interacting protein 24 |
|  |  |  |  | Solyc11g068800.1.1 | Transcription factor (E2F) |
| TTTCTACCCCTTTTTAGCCTA | 23 | 0 | - | Solyc01g110700.2.1 | Unknown Protein |
|  |  |  |  | Solyc02g077300.1.1 | Peroxidase 73 |
|  |  |  |  | Solyc03g031460.1.1 | Pentatricopeptide repeat-containing protein |
|  |  |  |  | Solyc03g058350.2.1 | Translation initiation factor |
|  |  |  |  | Solyc03g077870.1.1 | HAT-dimerisation domain-containing protein |
|  |  |  |  | Solyc03g114250.2.1 | Phosphoglycerate mutase family protein |
|  |  |  |  | Solyc03g122040.2.1 | COP1-Interacting Protein 7 |
|  |  |  |  | Solyc04g080480.1.1 | AMP-dependent synthetase and ligase |
|  |  |  |  | Solyc04g082920.2.1 | Chlorophyll a-b binding protein |
|  |  |  |  | Solyc05g008010.2.1 | 60S ribosomal protein L31 |
|  |  |  |  | Solyc05g011940.2.1 | Cytochrome P450 |
|  |  |  |  | Solyc06g083520.2.1 | GNL3L/Grn1 putative GTPase |
|  |  |  |  | Solyc08g078310.2.1 | DUF1639 |
|  |  |  |  | Solyc10g007080.2.1 | Aberrant lateral root formation 5 |
|  |  |  |  | Solyc12g098120.1.1 | Inositol oxygenase |
| TCCTATTACCCCCCTGAACTT | 22 | 0 | - | Solyc02g069660.2.1 | Acuolar protein sorting-associated protein 54 |
|  |  |  |  | solyc07g007200.2.1 | Ring finger protein 12 |
|  |  |  |  | solyc08g043170.2.1 | Gamma-glutamyl phosphate reductase |
|  |  |  |  | solyc10g084970.1.1 | Unknown Protein |
| TCTGTTCTATAGGCTCGTACC | 31 | 0 | - | Solyc01g057900.2.1 | Ubiquitin-protein ligase |
|  |  |  |  | Solyc02g062430.2.1 | D-lactate dehydrogenase 2 mitochondrial |
|  |  |  |  | Solyc04g080180.1.1 | Uncharacterized membrane protein |
|  |  |  |  | Solyc09g092180.2.1 | HAPp48 5 protein |
|  |  |  |  | Solyc10g084540.1.1 | Pentatricopeptide repeat-containing protein |
| ATGTCAGCCCAACTCCTCCTG | 141 | 0 | - | Solyc01g106190.2.1 | Os11g0167200 protein |
|  |  |  |  | Solyc02g082210.2.1 | Genomic DNA chromosome 3 P1 clone MOJ10 |
|  |  |  |  | Solyc04g005550.1.1 | Cc-nbs-lrr** |
|  |  |  |  | Solyc04g025160.2.1 | Arsenite ATPase transporter |
|  |  |  |  | solyc05g007480.1.1 | Phytochrome kinase substrate 1 |
|  |  |  |  | solyc06g075640.1.1 | Male sterility 5 family protein |
|  |  |  |  | solyc07g044800.2.1 | Nbs-lrr** |
|  |  |  |  | solyc09g064800.1.1 | Glycogen debranching enzyme |
|  |  |  |  | solyc10g074640.1.1 | Nbs** |
|  |  |  |  | solyc12g100360.1.1 | Calpain-like protein |
| CCCCCTCAACCTATGCCCGAA | 11 | 0 | - | Solyc01g110700.2.1 | Unknown Protein |
|  |  |  |  | Solyc11g013150.1.1 | GRAS family transcription factor |
| CCCGTGGACGTAGCCAATTTA | 20 | 0 | - | Solyc02g069050.1.1 | Unkown protein |
|  |  |  |  | Solyc05g013260.1.1 | Cc-nbs |
|  |  |  |  | Solyc08g016420.2.1 | Prefoldin subunit 6 |
| TAGAATGAGGATCGGAGTGTC | 25 | 0 | - | Solyc05g026210.1.1 | Glutathione S-transferase |
| CGCGGCGTCGTTGAGGAATGC | 245 | 0 | - | No hit |  |
| TGTGAGATACGTAGGCAGCCT | 25 | 0 | - | Solyc09g091600.2.1 | Tetratricopeptide-like helical |
| TGTGGCCTTAGAGCAAAGACT | 41 | 0 | - | Solyc02g092250.2.1 | Cytochrome P450 |
| ATCACTACTTCTCCCAATATC | 177 | 0 | - | Solyc01g018040.1.1 | MULE transposase-containing domain |
|  |  |  |  | Solyc01g081460.2.1 | tRNA (guanine-N(7)-)-methyltransferase |
|  |  |  |  | Solyc01g081620.2.1 | Vacuolar sorting protein |
|  |  |  |  | Solyc01g094340.2.1 | Acetyl-coA carboxylase |
|  |  |  |  | solyc01g111480.1.1 | Mutator-like transposase |
|  |  |  |  | solyc02g078670.2.1 | COP1-Interacting ProteinI 7 (CIP7) |
|  |  |  |  | solyc02g087190.1.1 | Peroxidase 65 |
|  |  |  |  | solyc02g093580.2.1 | Pectate lyase |
|  |  |  |  | solyc03g083960.2.1 | Trehalose 6-phosphate phosphatase |
|  |  |  |  | solyc03g112690.1.1 | Histone-lysine N-methyltransferase |
|  |  |  |  | solyc03g116950.1.1 | PEARLI 4 protein |
|  |  |  |  | solyc03g118640.2.1 | D-tagatose-1,6-bisphosphate aldolase |
|  |  |  |  | solyc04g040110.2.1 | Kinesin-like protein |
|  |  |  |  | solyc04g079820.2.1 | Inositol 5-phosphatase 4 |
|  |  |  |  | solyc05g018570.2.1 | 26S protease regulatory subunit 8 |
|  |  |  |  | solyc05g052240.2.1 | Chalcone--flavonone isomerase |
|  |  |  |  | solyc06g076340.2.1 | mRNA binding protein Pumilio 2 |
|  |  |  |  | solyc06g082530.1.1 | GRAS family transcription factor |
|  |  |  |  | solyc07g006610.2.1 | Serine/threonine protein kinase |
|  |  |  |  | solyc07g039500.2.1 | Group II intron splicing factor CRS1-like |
|  |  |  |  | solyc08g075790.2.1 | Vacuolar sorting protein |
|  |  |  |  | solyc08g077150.2.1 | Protein phosphatase 2C containing protein |
|  |  |  |  | solyc08g080960.2.1 | AT-hook motif nuclear localized protein 1 |
|  |  |  |  | solyc08g081250.2.1 | Aminopeptidase N |
|  |  |  |  | solyc09g075550.2.1 | Cellulose synthase-like D6 |
|  |  |  |  | solyc09g091840.2.1 | Glutathione-disulfide reductase |
|  |  |  |  | solyc10g007200.2.1 | Beta-1 3-galactosyltransferase 6 |
|  |  |  |  | solyc10g018780.1.1 | Squamosa promoter binding protein 1 |
|  |  |  |  | solyc10g080510.1.1 | Receptor-like kinase |
|  |  |  |  | solyc10g081470.1.1 | Peptidase M50 family protein |
|  |  |  |  | solyc11g005230.1.1 | Sensitivity to red light reduced protein 1 |
|  |  |  |  | solyc12g038340.1.1 | Translation initiation factor eIF-2B |
| AGAGAAAAAGGCCAACGAATT | 13 | 0 | - | Solyc01g100000.2.1 | F-box protein PP2-B1 |
|  |  |  |  | Solyc02g070260.2.1 | Protein phosphatase 1 regulatory subunit 7 |
|  |  |  |  | Solyc02g078230.1.1 | Glucan synthase like 1 |
|  |  |  |  | Solyc03g006290.2.1 | DUF599 family protein |
|  |  |  |  | Solyc03g044100.2.1 | Peroxidase 5 |
|  |  |  |  | Solyc04g015760.2.1 | Protein N-terminal glutamine amidohydrolase |
|  |  |  |  | Solyc06g005590.2.1 | Telomere repeat-binding protein 5 |
|  |  |  |  | Solyc06g051980.2.1 | E3 ubiquitin-protein ligase sina |
|  |  |  |  | Solyc06g066650.2.1 | Pollen-specific kinase |
|  |  |  |  | Solyc06g073490.1.1 | Unknown Protein |
|  |  |  |  | Solyc06g076270.2.1 | Telomere repeat-binding protein 5 |
|  |  |  |  | Solyc08g007180.2.1 | Light harvesting-like protein 3 |
|  |  |  |  | Solyc09g007890.1.1 | Phenylalanine ammonia-lyase |
|  |  |  |  | Solyc09g025310.2.1 | NAC domain protein |
|  |  |  |  | Solyc10g085150.1.1 | Undecaprenyl pyrophosphate synthase |
| TTCTACCCCTTTTCGGCCTAC | 33 | 0 | - | Solyc01g091840.2.1 | 5'-phosphosulfate transmembrane transporter |
|  |  |  |  | Solyc01g110700.2.1 | Unknown Protein |
|  |  |  |  | Solyc03g122040.2.1 | COP1-Interacting Protein |
|  |  |  |  | Solyc06g083520.2.1 | Nucleolar GTP-binding protein 2 |
|  |  |  |  | Solyc09g090920.2.1 | C2 domain-containing protein |
|  |  |  |  | Solyc10g007080.2.1 | Aberrant lateral root formation 5 |
|  |  |  |  | Solyc11g069500.1.1 | Auxin response factor 16 |
|  |  |  |  | Solyc12g098120.1.1 | Inositol oxygenase |
|  |  |  |  | Solyc12g099910.1.1 | Chromodomain helicase DNA binding protein 2 |
| TACTAAGGTCCTATTACCCCC | 28 | 0 | - | Solyc01g090330.1.1 | Unknown Protein |
|  |  |  |  | Solyc10g007080.2.1 | Aberrant lateral root formation 5 |
|  |  |  |  | Solyc12g017390.1.1 | Protein kinase |
| ACAACATCATCGAACAACCTT | 20 | 0 | - | Solyc00g020540.1.1 | Aminotransferase-like protein |
|  |  |  |  | Solyc01g007170.2.1 | Protein unc-13 homolog C |
|  |  |  |  | Solyc01g067150.1.1 | Unknown Protein |
|  |  |  |  | Solyc01g100450.1.1 | Pentatricopeptide repeat-containing protein |
|  |  |  |  | Solyc02g032200.2.1 | Tir-lrr |
|  |  |  |  | Solyc02g036270.2.1 | Cc-nbs-lrr** |
|  |  |  |  | Solyc02g082140.2.1 | Unknown Protein |
|  |  |  |  | Solyc02g089890.2.1 | NAD-dependent epimerase/dehydratase |
|  |  |  |  | Solyc03g093640.2.1 | Polycomb group protein EMBRYONIC FLOWER 2 |
|  |  |  |  | Solyc03g094100.1.1 | Nbs** |
|  |  |  |  | Solyc04g016550.2.1 | Folylpolyglutamate synthase |
|  |  |  |  | Solyc04g049010.2.1 | exoribonuclease 2 |
|  |  |  |  | Solyc04g049440.1.1 | WPP domain-associated protein |
|  |  |  |  | Solyc05g051580.2.1 | Phototropic-responsive NPH3 family protein |
|  |  |  |  | Solyc06g073110.2.1 | Legume lectin beta domain |
|  |  |  |  | Solyc07g044800.2.1 | Nbs-lrr |
|  |  |  |  | Solyc09g008350.2.1 | Amino acid binding protein |
|  |  |  |  | Solyc09g014390.1.1 | Alpha 1 4-galactosyltransferase |
|  |  |  |  | Solyc09g075710.1.1 | Gibberellin receptor GID1L2 |
|  |  |  |  | Solyc09g097850.1.1 | Cysteine proteinase inhibitor |
|  |  |  |  | Solyc09g097880.2.1 | DNA topoisomerase |
|  |  |  |  | Solyc09g098050.2.1 | Ankyrin repeat-containing protein At5g02620 |
|  |  |  |  | Solyc10g084640.1.1 | N-acetyltransferase |
|  |  |  |  | Solyc12g014540.1.1 | Lysine-specific demethylase 5C |
| CACCCGATCCATATTCTATCC | 16 | 0 | - | Solyc01g096120.2.1 | Dof zinc finger protein 2 |
|  |  |  |  | Solyc03g113340.2.1 | Nodulin-like protein |
|  |  |  |  | Solyc05g005010.2.1 | Carboxyl-terminal proteinase |
|  |  |  |  | Solyc05g026490.2.1 | Phosphoglucomutase/phosphomannomutase family protein |
|  |  |  |  | Solyc09g010220.2.1 | Lysosomal Pro-X carboxypeptidase |
|  |  |  |  | Solyc11g064850.1.1 | Unknown |
| TTTTCTACCCCTTTTCGGCCT | 228 | 0 | - | Solyc01g111180.2.1 | Unknown Protein |
|  |  |  |  | Solyc02g073580.1.1 | bZIP transcription factor |
|  |  |  |  | Solyc02g078330.2.1 | Pentatricopeptide repeat-containing protein |
|  |  |  |  | Solyc03g116680.2.1 | Methyl binding domain protein |
|  |  |  |  | Solyc03g122040.2.1 | COP1-Interacting Protein 7 |
|  |  |  |  | Solyc05g010280.2.1 | LAG1 longevity assurance homolog 6 |
|  |  |  |  | Solyc06g062430.2.1 | Inositol oxygenase |
|  |  |  |  | Solyc08g013740.2.1 | Dehydration-responsive family protein |
|  |  |  |  | Solyc09g048980.2.1 | Phototropic-responsive NPH3 family protein |
|  |  |  |  | Solyc09g090920.2.1 | C2 domain-containing protein |
|  |  |  |  | Solyc10g007080.2.1 | Aberrant lateral root formation 5 |
|  |  |  |  | Solyc10g008900.2.1 | Disease resistance response |
|  |  |  |  | Solyc11g012760.1.1 | TRNA (Guanine-N1-)-methyltransferase |
|  |  |  |  | Solyc11g068490.1.1 | Unknown Protein |
|  |  |  |  | Solyc12g098120.1.1 | Inositol oxygenase |
|  |  |  |  | Solyc12g099910.1.1 | Chromodomain helicase DNA binding protein 2 |
| TTTTCTACCCCTTTTCAGCCT | 20 | 0 | - | Solyc01g080760.2.1 | Unknown Protein |
|  |  |  |  | Solyc01g095480.2.1 | Ribonuclease PH |
|  |  |  |  | Solyc01g106950.2.1 | Unknown Protein |
|  |  |  |  | Solyc01g111990.2.1 | Alanyl-tRNA synthetase |
|  |  |  |  | Solyc02g067400.1.1 | Glutaredoxin family protein |
|  |  |  |  | Solyc02g073580.1.1 | bZIP transcription factor |
|  |  |  |  | Solyc02g077300.1.1 | Peroxidase 73 |
|  |  |  |  | Solyc02g084820.2.1 | Glycosyl transferase group 1 |
|  |  |  |  | Solyc03g031460.1.1 | Pentatricopeptide repeat-containing protein |
|  |  |  |  | Solyc03g114250.2.1 | Phosphoglycerate mutase family protein |
|  |  |  |  | Solyc03g116950.1.1 | PEARLI 4 protein |
|  |  |  |  | Solyc03g122040.2.1 | COP1-Interacting ProteinI 7 |
|  |  |  |  | Solyc05g008010.2.1 | 60S ribosomal protein L31 |
|  |  |  |  | Solyc06g053890.1.1 | Fatty acid elongase 3-ketoacyl-CoA synthase |
|  |  |  |  | Solyc07g065490.2.1 | Dek protein |
|  |  |  |  | Solyc08g075930.2.1 | Kinesin-like |
|  |  |  |  | Solyc08g080860.2.1 | Unknown Protein |
|  |  |  |  | Solyc09g048980.2.1 | Phototropic-responsive NPH3 family protein |
|  |  |  |  | Solyc10g007080.2.1 | Aberrant lateral root formation 5 |
|  |  |  |  | Solyc11g006300.1.1 | 3-oxo-5-alpha-steroid 4-dehydrogenase |
|  |  |  |  | Solyc11g008670.1.1 | Unknown Protein |
|  |  |  |  | Solyc12g098120.1.1 | Inositol oxygenase |
|  |  |  |  | Solyc12g099910.1.1 | Chromodomain helicase DNA binding protein |
| ATAAAACTGCTCCCTCTCTTC | 13 | 0 | - | Solyc01g105300.2.1 | Metacaspase |
|  |  |  |  | Solyc02g071490.2.1 | 1-aminocyclopropane-1-carboxylate oxidase 1 |
|  |  |  |  | Solyc02g072290.1.1 | Subtilisin-like protease |
|  |  |  |  | Solyc02g086980.2.1 | Unknown Protein |
|  |  |  |  | Solyc04g055030.1.1 | Iron sulfur subunit of succinate dehydrogenase |
|  |  |  |  | Solyc04g072850.2.1 | Alpha-L-arabinofuranosidase/beta-D-xylosidase |
|  |  |  |  | Solyc04g078830.2.1 | HO complex subunit 5 homolog |
|  |  |  |  | Solyc04g079170.2.1 | Unknown Protein |
|  |  |  |  | Solyc05g006760.2.1 | Tetratricopeptide repeat protein 38 |
|  |  |  |  | Solyc05g007170.2.1 | Cc-nbs-lrr |
|  |  |  |  | Solyc11g018660.1.1 | NAC domain protein |
|  |  |  |  | Solyc11g044450.1.1 | Chaperone protein dnaJ 2 |
|  |  |  |  | Solyc12g013690.1.1 | Monooxygenase FAD-binding protein |
|  |  |  |  | Solyc12g098160.1.1 | Always early protein 3 |
| TACGCAGCAGATCTCCACGAC | 134 | 0 | - | Solyc01g103080.2.1 | ATP-dependent RNA helicase DOB1 |
|  |  |  |  | Solyc02g036270.2.1 | Cc-nbs-lrr** |
|  |  |  |  | Solyc02g086080.2.1 | Alpha/beta hydrolase fold |
|  |  |  |  | Solyc03g094100.1.1 | Nbs** |
|  |  |  |  | Solyc03g123690.1.1 | Ring H2 finger protein |
|  |  |  |  | Solyc08g067300.1.1 | F-box family protein |
| TTCTCCTTCCTTGTCTATCCC | 0 | 138 | - | Solyc01g005580.2.1 | Unknown Protein |
|  |  |  |  | Solyc01g008590.1.1 | Zinc transporter protein |
|  |  |  |  | Solyc01g108990.2.1 | Unknown Protein |
|  |  |  |  | Solyc02g069600.2.1 | Cytochrome P450 |
|  |  |  |  | Solyc02g077970.2.1 | Late embryo abundance protein |
|  |  |  |  | Solyc02g093510.2.1 | Unknown Protein |
|  |  |  |  | Solyc02g093830.2.1 | Glucose-6-phosphate 1-dehydrogenase |
|  |  |  |  | Solyc03g031570.2.1 | Dopamine beta-monooxygenase |
|  |  |  |  | Solyc03g098430.2.1 | Xyloglucan endotransglucosylase/hydrolase 5 |
|  |  |  |  | Solyc03g114720.2.1 | Transcription factor BIM2 |
|  |  |  |  | Solyc04g071660.2.1 | Unknown Protein |
|  |  |  |  | Solyc04g076790.2.1 | Serine hydroxymethyltransferase |
|  |  |  |  | Solyc05g013500.2.1 | Glycosyltransferase-like protein |
|  |  |  |  | Solyc05g052410.1.1 | Ethylene-responsive transcription factor 1 |
|  |  |  |  | Solyc05g054010.2.1 | Cc-nbs-lrr |
|  |  |  |  | Solyc06g050180.1.1 | Unknown Protein |
|  |  |  |  | Solyc06g065790.1.1 | Serine/threonine-protein kinase 3 |
|  |  |  |  | Solyc06g069370.2.1 | Transcription factor protein |
|  |  |  |  | Solyc06g073470.2.1 | iotin synthase |
|  |  |  |  | Solyc06g074390.2.1 | Fatty acyl coA reductase |
|  |  |  |  | Solyc06g074980.2.1 | 26S protease regulatory subunit 6B homolog |
|  |  |  |  | Solyc07g054990.1.1 | Unknown Protein |
|  |  |  |  | Solyc08g080690.2.1 | Methyl-CpG DNA binding protein |
|  |  |  |  | Solyc09g031610.2.1 | WD repeat containing protein |
|  |  |  |  | Solyc09g075830.2.1 | Time for coffee |
|  |  |  |  | Solyc09g091930.2.1 | Ubiquitin carboxyl-terminal hydrolase |
|  |  |  |  | Solyc10g050190.1.1 | Unknown Protein |
|  |  |  |  | Solyc11g021290.1.1 | Ycf1 |
|  |  |  |  | Solyc11g051060.1.1 | GDSL esterase/lipase 2 |
|  |  |  |  | Solyc11g064920.1.1 | Dihydropyrimidinase |
|  |  |  |  | Solyc11g072150.1.1 | Nuclear transcription factor Y subunit C-1 |
|  |  |  |  | Solyc11g072480.1.1 | Senescence-associated protein |
|  |  |  |  | Solyc11g072710.1.1 | Serine/threonine protein kinase |
|  |  |  |  | Solyc12g014010.1.1 | Glucosyltransferase |
|  |  |  |  | Solyc12g035250.1.1 | Zinc finger CCCH domain-containing protein 41 |
| CCCGAAATCCCAGAGACACAC | 0 | 35 | - | Slyc01g006000.2.1 | GPI mannosyltransferase 2 |
|  |  |  |  | olyc01g105540.2.1 | 2-oxoglutarate/malate translocator |
|  |  |  |  | Solyc01g110700.2.1 | Unknown Protein |
|  |  |  |  | Solyc10g007080.2.1 | Aberrant lateral root formation 5 |
| TAATGCTGGAACTTGCACACC | 0 | 76 | - | Solyc01g095720.2.1 | Lipase |
|  |  |  |  | Solyc02g087290.2.1 | Alpha-mannosidase-like protein |
|  |  |  |  | Solyc03g115540.1.1 | BHLH transcription factor |
|  |  |  |  | Solyc04g054900.2.1 | Unknown protein |
|  |  |  |  | Solyc06g054600.2.1 | Zinc finger CCCH domain-containing protein 58 |
|  |  |  |  | Solyc08g065490.2.1 | Serine hydroxymethyltransferase |
|  |  |  |  | Solyc09g083410.2.1 | Amidase hydantoinase |
|  |  |  |  | Solyc12g006670.1.1 | Sel1 domain protein repeat-containing protein |
| TTGCAGTTCTGGATAAGTGGC | 0 | 167 | - | Solyc00g014960.1.1 | F-box family protein |
|  |  |  |  | Solyc01g088760.2.1 | Cytochrome P450 |
|  |  |  |  | Solyc01g094400.2.1 | Actin depolymerizing factor 10 |
|  |  |  |  | Solyc06g054600.2.1 | Zinc finger CCCH domain-containing protein 58 |
|  |  |  |  | Solyc09g090110.2.1 | Actin depolymerizing factor 6 |
| TTTTCTACCCCTTTTTGGCCT | 0 | 15 | - | Solyc01g005920.2.1 | Amino acid permease |
|  |  |  |  | Solyc01g102740.2.1 | NAC domain protein |
|  |  |  |  | Solyc01g110700.2.1 | Unknown Protein |
|  |  |  |  | Solyc02g078330.2.1 | Pentatricopeptide repeat-containing protein |
|  |  |  |  | Solyc02g092940.2.1 | Receptor like kinase |
|  |  |  |  | Solyc03g031460.1.1 | Pentatricopeptide repeat-containing protein |
|  |  |  |  | Solyc03g111130.1.1 | Malate synthase |
|  |  |  |  | Solyc03g122040.2.1 | COP1-Interacting ProteinI 7 |
|  |  |  |  | Solyc05g005580.2.1 | Unknown Protein |
|  |  |  |  | Solyc05g010280.2.1 | LAG1 longevity assurance homolog 6 |
|  |  |  |  | Solyc05g011940.2.1 | Cytochrome P450 |
|  |  |  |  | Solyc06g062430.2.1 | Inositol oxygenase |
|  |  |  |  | Solyc08g013740.2.1 | Dehydration-responsive family protein |
|  |  |  |  | Solyc08g075850.2.1 | 50S ribosomal protein L24 |
|  |  |  |  | Solyc08g082170.2.1 | polygalacturonase |
|  |  |  |  | Solyc09g008990.2.1 | Cellulose synthase |
|  |  |  |  | Solyc09g048980.2.1 | Phototropic-responsive NPH3 family protein |
|  |  |  |  | Solyc09g090210.2.1 | Serine/threonine protein kinase |
|  |  |  |  | Solyc09g090920.2.1 | C2 domain-containing protein |
|  |  |  |  | Solyc10g007080.2.1 | Aberrant lateral root formation 5 |
|  |  |  |  | Solyc10g008900.2.1 | Disease resistance response |
|  |  |  |  | Solyc11g005070.1.1 | Amino acid permease |
|  |  |  |  | Solyc11g011780.1.1 | mRNA decay NMD3 family protein |
|  |  |  |  | Solyc11g012760.1.1 | TRNA (Guanine-N1-)-methyltransferase |
|  |  |  |  | Solyc11g045450.1.1 | B3 domain-containing protein |
|  |  |  |  | Solyc12g010360.1.1 | H-ATPase |
|  |  |  |  | Solyc12g095940.1.1 | Unknown Protein |
|  |  |  |  | Solyc12g098120.1.1 | Inositol oxygenase |
| ATCCAGGGCAACAAGAAACTT | 0 | 50 | - | Solyc01g044480.2.1 | Elongation factor P |
|  |  |  |  | Solyc01g060150.2.1 | Unknown Protein |
|  |  |  |  | Solyc01g102880.1.1 | Tir-nbs-lrr |
|  |  |  |  | Solyc02g032240.1.1 | Nbs** |
|  |  |  |  | Solyc02g063450.2.1 | Hypothetical YFW family protein 5 |
|  |  |  |  | Solyc02g083450.2.1 | Aspartic proteinase-like protein 1 |
|  |  |  |  | Solyc03g006280.2.1 | Unknown Protein |
|  |  |  |  | Solyc03g032140.2.1 | Heterogeneous nuclear ribonucleoprotein H1 |
|  |  |  |  | Solyc06g063370.2.1 | Chlorophyll a-b binding protein 1A |
|  |  |  |  | Solyc07g008040.2.1 | WD-40 repeat protein-like |
|  |  |  |  | Solyc07g042000.2.1 | Sodium/calcium exchanger protein |
|  |  |  |  | Solyc07g044870.2.1 | Polygalacturonase |
|  |  |  |  | Solyc07g065240.1.1 | Receptor like kinase |
|  |  |  |  | Solyc08g007630.1.1 | Cc-nbs-lrr |
|  |  |  |  | Solyc08g042130.2.1 | Phox domain-containing protein |
|  |  |  |  | Solyc08g063050.2.1 | Cell division protease ftsH homolog |
|  |  |  |  | Solyc11g007840.1.1 | Glycosyltransferase family protein 1 |
|  |  |  |  | Solyc11g072020.1.1 | Ubiquitin |
| TCTCAAGTCATCCCACTCATT | 0 | 120 | - | No hit |  |
| TTCTTGACCTTGTAAGACCCC | 21 | 6 | 0.1 | Solyc01g090490.1.1 | Mitochodrial transcription termination factor |
|  |  |  |  | Solyc02g077560.2.1 | Auxin response factor 3 |
|  |  |  |  | Solyc03g019840.2.1 | Plastid DNA-binding protein |
|  |  |  |  | Solyc03g118290.2.1 | Auxin response factor 2 |
|  |  |  |  | Solyc04g078050.2.1 | Transcription initiation factor TFIID component |
|  |  |  |  | Solyc08g074440.2.1 | Pre-mRNA-splicing factor CWC21 |
|  |  |  |  | Solyc10g005210.2.1 | DNA-3-methyladenine glycosylase I |
|  |  |  |  | Solyc11g069190.1.1 | Auxin response factor 4 |
| ATGTTAGGCCAACTCCTCCAG | 314 | 223 | -0.6 | Solyc02g036270.2.1 | Cc-nbs-lrr** |
|  |  |  |  | Solyc03g116260.2.1 | Gibberellin 2-beta-dioxygenase 7 |
|  |  |  |  | Solyc03g120720.2.1 | Protein disulfide isomerase L-3b |
|  |  |  |  | Solyc04g076230.2.1 | AP-2 complex subunit mu |
|  |  |  |  | Solyc08g005240.1.1 | Lysine-specific demethylase 5B |
|  |  |  |  | Solyc12g057130.1.1 | Nbs |
| TCCAGAAATTGTCGCCTTGGA | 303 | 146 | -0.2 | Solyc01g088640.2.1 | Ring finger protein 12 |
|  |  |  |  | Solyc01g105330.2.1 | Methyl binding domain protein |
|  |  |  |  | Solyc01g110700.2.1 | Unknown Protein |
|  |  |  |  | Solyc02g036270.2.1 | Cc-nbs-lrr |
|  |  |  |  | Solyc02g067480.2.1 | Pyridine nucleotide-disulfide oxidoreductase |
|  |  |  |  | Solyc03g005130.1.1 | Receptor-like protein kinase |
|  |  |  |  | Solyc03g053080.1.1 | Kinesin like protein |
|  |  |  |  | Solyc03g093100.1.1 | Mutator-like transposase |
|  |  |  |  | Solyc03g094100.1.1 | Nbs |
|  |  |  |  | Solyc06g083390.2.1 | RPM1 interacting protein 4 transcript 2 |
|  |  |  |  | Solyc07g045180.2.1 | CONSTANS-like zinc finger protein |
|  |  |  |  | Solyc08g016290.1.1 | Mutator-like transposase |
|  |  |  |  | Solyc09g090740.2.1 | ATP-dependent RNA helicase |
| CAAGGCGACAATTTCTGGAAT | 176 | 59 | 0.3 | Solyc01g095690.2.1 | tatricopeptide repeat-containing protein |
|  |  |  |  | Solyc02g089670.2.1 | Leaf senescence protein-like |
|  |  |  |  | Solyc02g091390.2.1 | Little protein 1 |
|  |  |  |  | Solyc03g098220.2.1 | Serine palmitoyltransferase |
|  |  |  |  | Solyc06g064910.2.1 | T-complex protein 11 |
|  |  |  |  | Solyc08g081260.1.1 | Tir-nbs |
|  |  |  |  | Solyc12g021170.1.1 | Electron-transfer flavoprotein |
| TGAAGGTCCGAGGTTGAGGTT | 969 | 456 | -0.1 | Solyc05g009850.2.1 | Gc-rich sequence DNA-binding factor |
|  |  |  |  | Solyc10g008420.2.1 | Unknown Protein |
|  |  |  |  | Solyc10g086250.1.1 | MYB transcription factor |
| ACTTCATGTTGTCTTTGGGCC | 121 | 109 | -0.8 | Solyc03g095680.1.1 | SNF2 helicase |
|  |  |  |  | Solyc04g050910.1.1 | Unknown Protein |
|  |  |  |  | Solyc07g044930.2.1 | ATP-dependent RNA helicase A |
|  |  |  |  | Solyc11g042780.1.1 | Transposase |
|  |  |  |  | Solyc12g006910.1.1 | Unknown Protein |
|  |  |  |  | Solyc12g008650.1.1 | Inositol oxygenase |
| TACGCAGCAGATCTCCACGAC | 134 | 75 | -0.3 | Solyc01g103080.2.1 | TP-dependent RNA helicase DOB1 |
|  |  |  |  | Solyc02g036270.2.1 | Cc-nbs-lrr* |
|  |  |  |  | Solyc02g086080.2.1 | Alpha/beta hydrolase fold |
|  |  |  |  | Solyc03g094100.1.1 | Nbs* |
|  |  |  |  | Solyc03g123690.1.1 | Ring H2 finger protein |
|  |  |  |  | Solyc07g044800.2.1 | Nbs-lrr |
|  |  |  |  | Solyc08g067300.1.1 | F-box family protein |

*DE is differential expression values where are only presented for ta-siRNAs found in both WT and *7B-1*. DE values were calculated as log_2_-fold changes of the expression (*7B-1*/WT). Negative and positive values mean up- and down-regulation of the expression in 7B-1, respectively. ** indicates targets which have identified from multiple loci.

**Table S6**. List of the identified *TAS3-*derived ta-siRNAs from WT library.

| **Locus** | **Sequence** | **Size** | **Abundance** | **Start** | **Stop** | **Strand** |
| --- | --- | --- | --- | --- | --- | --- |
| TAS3_DV105041:1 | TTCCTCTACCTACCCCATTCT | 21 | 57 | 337 | 357 | + |
| TAS3_DV105041:1 | TTCTTGACCTTGTAAGACCCC * | 23 | 21 | 378 | 398 | + |
| TAS3_DV105041:1 | ATGGGGTAGGTAGAGGAACGA | 21 | 19 | 334 | 354 | - |
| TAS3_DV105041:1 | TAAGCTTTGTTTGGTCTCCTTC | 22 | 13 | 462 | 483 | + |
| TAS3_DV105041:1 | TTCTTGACCTTGTAAGACCTT ** | 24 | 10 | 357 | 377 | + |
| TAS3_DV105041:1 | TTTTTTGACTTGTTGCCTTTC | 21 | 9 | 315 | 335 | + |
| TAS3_DV105041:1 | TCGTTCCTCTACCTACCCCATT | 22 | 7 | 334 | 355 | + |
| TAS3_DV105041:1 | TCTTGACCTTGTAAGACCCCG | 21 | 7 | 379 | 399 | + |
| TAS3_DV105041:1 | TTGACCTTGTAAGACCCCGTG | 21 | 7 | 381 | 401 | + |
| TAS3_DV105041:1 | TTGACCTTGTAAGACCCCGTGT | 22 | 6 | 381 | 402 | + |
| TAS3_DV105041:1 | TTTGGTCTCCTTCTTCTTTCCT | 22 | 6 | 471 | 492 | + |
| TAS3_DV105041:1 | TAAGACCCCGTGTTATCTCTT | 21 | 5 | 390 | 410 | + |
| TAS3_DV105041:1 | AAGCTTTGTTTGGTCTCCTTC | 21 | 5 | 463 | 483 | + |
| TAS3_DV105041:1 | AGCTTTGTTTGGTCTCCTTCTT | 22 | 5 | 464 | 485 | + |
| TAS3_DV105041:1 | TAATCTCGGTGCTATCCTAC | 20 | 4 | 251 | 270 | + |
| TAS3_DV105041:1 | TCAGGTAGGATAGCACCGAGAT | 22 | 4 | 253 | 274 | - |
| TAS3_DV105041:1 | TCTCTTTTTTGACTTGTTGCCT | 22 | 4 | 311 | 332 | + |
| TAS3_DV105041:1 | TGTTGCCTTTCGTTCCTCTAC | 21 | 4 | 325 | 345 | + |
| TAS3_DV105041:1 | TGTAAGACCCCGTGTTATCTC | 21 | 4 | 388 | 408 | + |
| TAS3_DV105041:1 | AGAAGGAGACCAAACAAAGCT | 21 | 4 | 464 | 484 | - |
| TAS3_DV105041:1 | TTTGTTTGGTCTCCTTCTTCT | 21 | 4 | 467 | 487 | + |
| TAS3_DV105041:1 | TCCTCCTTCCTTGTCTATCCC | 21 | 4 | 505 | 525 | + |
| TAS3_DV105041:1 | TTTTGACTTGTTGCCTTTCGTT | 22 | 3 | 317 | 338 | + |
| TAS3_DV105041:1 | ACTTGTTGCCTTTCGTTCCT | 20 | 3 | 322 | 341 | + |
| TAS3_DV105041:1 | CTTGTTGCCTTTCGTTCCTCT | 21 | 3 | 323 | 343 | + |
| TAS3_DV105041:1 | TTGCCTTTCGTTCCTCTACCT | 21 | 3 | 327 | 347 | + |
| TAS3_DV105041:1 | TTTCGTTCCTCTACCTACCCC | 21 | 3 | 332 | 352 | + |
| TAS3_DV105041:1 | GTTCCTCTACCTACCCCATTC | 21 | 3 | 336 | 356 | + |
| TAS3_DV105041:1 | AGAATGGGGTAGGTAGAGGAA | 21 | 3 | 337 | 357 | - |
| TAS3_DV105041:1 | TTCCTCTACCTACCCCATTCTT | 22 | 3 | 337 | 358 | + |
| TAS3_DV105041:1 | TTCTTGACCTTGTAAGACCT | 20 | 3 | 357 | 376 | + |
| TAS3_DV105041:1 | ATAACACGGGGTCTTACAAGGT | 22 | 3 | 384 | 405 | - |
| TAS3_DV105041:1 | TCTTACGTCATGACTTCTTCA | 21 | 3 | 439 | 459 | + |
| TAS3_DV105041:1 | AAGCTTTGTTTGGTCTCCTTCT | 22 | 3 | 463 | 484 | + |
| TAS3_DV105041:1 | TATCCCTCCTGAGCTGTTGATT | 22 | 3 | 520 | 541 | + |
| TAS3_DV105041:1 | CATGTTTAGTGGTCCTTCTTA | 21 | 2 | 174 | 194 | + |
| TAS3_DV105041:1 | TGAGGATGCGACACTCATCGT | 21 | 2 | 227 | 247 | - |
| TAS3_DV105041:1 | TTAAGATGAGGATGCGACACT | 21 | 2 | 233 | 253 | - |
| TAS3_DV105041:1 | TCCTCATCTTAATCTCGGTGCT | 22 | 2 | 242 | 263 | + |
| TAS3_DV105041:1 | CAGGTAGGATAGCACCGAGAT | 21 | 2 | 253 | 273 | - |
| TAS3_DV105041:1 | TTGTTGCCTTTCGTTCCTCTACC | 23 | 2 | 324 | 346 | + |
| TAS3_DV105041:1 | TCGTTCCTCTACCTACCCCAT | 21 | 2 | 334 | 354 | + |
| TAS3_DV105041:1 | TCCTCTACCTACCCCATTCTT | 21 | 2 | 338 | 358 | + |
| TAS3_DV105041:1 | AAGAATGGGGTAGGTAGAGGA | 21 | 2 | 338 | 358 | - |
| TAS3_DV105041:1 | CTCTACCTACCCCATTCTTCTT | 22 | 2 | 340 | 361 | + |
| TAS3_DV105041:1 | TACAAGGTCAAGAAGAATGGGG | 22 | 2 | 349 | 370 | - |
| TAS3_DV105041:1 | TTACAAGGTCAAGAAAAGGTCT | 22 | 2 | 371 | 392 | - |
| TAS3_DV105041:1 | TGACCTTGTAAGACCCCGTGT | 21 | 2 | 382 | 402 | + |
| TAS3_DV105041:1 | TTACGTCATGACTTCTTCATG | 21 | 2 | 441 | 461 | + |
| TAS3_DV105041:1 | TTTGGTCTCCTTCTTCTTTCC | 21 | 2 | 471 | 491 | + |
| TAS3_DV105041:1 | TTGGTCTCCTTCTTCTTTCC | 20 | 2 | 472 | 491 | + |
| TAS3_DV105041:1 | TAGGAAGACGAAGAGAAAGCC | 21 | 1 | 206 | 226 | - |
| TAS3_DV105041:1 | TGCGACACTCATCGTTAGGAAG | 22 | 1 | 220 | 241 | - |
| TAS3_DV105041:1 | CATCCTCATCTTAATCTCGGTGCT | 24 | 1 | 240 | 263 | + |
| TAS3_DV105041:1 | ATCCTCATCTTAATCTCGGTGCT | 23 | 1 | 241 | 263 | + |
| TAS3_DV105041:1 | AATCTCGGTGCTATCCTACCT | 21 | 1 | 252 | 272 | + |
| TAS3_DV105041:1 | TCCTACCTGAGCTTTTTCTCACC | 23 | 1 | 265 | 287 | + |
| TAS3_DV105041:1 | CGGTGAGAAAAAGCTCAGGTAGGATAG | 27 | 1 | 262 | 288 | - |
| TAS3_DV105041:1 | TTTTTGACTTGTTGCCTTTCGT | 22 | 1 | 316 | 337 | + |
| TAS3_DV105041:1 | AGGAACGAAAGGCAACAAGTC | 21 | 1 | 321 | 341 | - |
| TAS3_DV105041:1 | TTGTTGCCTTTCGTTCCTCTA | 21 | 1 | 324 | 344 | + |
| TAS3_DV105041:1 | TTTCGTTCCTCTACCTACCC | 20 | 1 | 332 | 351 | + |
| TAS3_DV105041:1 | CTTTCGTTCCTCTACCTACCCC | 22 | 1 | 331 | 352 | + |
| TAS3_DV105041:1 | TTTCGTTCCTCTACCTACCCCA | 22 | 1 | 332 | 353 | + |
| TAS3_DV105041:1 | TTCGTTCCTCTACCTACCCCA | 21 | 1 | 333 | 353 | + |
| TAS3_DV105041:1 | CAAGAAGAATGGGGTAGGTAG | 21 | 1 | 342 | 362 | - |
| TAS3_DV105041:1 | TACCCCATTCTTCTTGACCTTG | 22 | 1 | 347 | 368 | + |
| TAS3_DV105041:1 | CAAGGTCAAGAAGAATGGGGT | 21 | 1 | 348 | 368 | - |
| TAS3_DV105041:1 | TTCTTGACCTTGTAAGACCCCG | 22 | 1 | 378 | 399 | + |
| TAS3_DV105041:1 | TGACCTTGTAAGACCCCGTGTT | 22 | 1 | 382 | 403 | + |
| TAS3_DV105041:1 | TTTGTTTGGTCTCCTTCTTCTT | 22 | 1 | 467 | 488 | + |
| TAS3_DV105041:1 | TTTGGTCTCCTTCTTCTTTC | 20 | 1 | 471 | 490 | + |
| TAS3_DV105041:1 | CTCCTTCCTTGTCTATCCCT | 20 | 1 | 507 | 526 | + |
| TAS3_DV105041:1 | TATCCCTCCTGAGCTGTTGAT | 21 | 1 | 520 | 540 | + |

*, ** show D7 and D8 tasi*ARFs*, respectively.

**Table S7**. List of the identified *TAS3*-derived ta-siRNAs from *7B-1* library.

| **Locus** | **Sequence** | **Size** | **Abundance** | **Start** | **Stop** | **Strand** |
| --- | --- | --- | --- | --- | --- | --- |
| TAS3_DV105041:1 | TTCCTCTACCTACCCCATTCT | 21 | 25 | 337 | 357 | + |
| TAS3_DV105041:1 | TAAGCTTTGTTTGGTCTCCTTC | 22 | 13 | 462 | 483 | + |
| TAS3_DV105041:1 | TTCTTGACCTTGTAAGACCCC* | 22 | 6 | 378 | 398 | + |
| TAS3_DV105041:1 | TCTTGACCTTGTAAGACCCCG | 21 | 6 | 379 | 399 | + |
| TAS3_DV105041:1 | CTCTACCTACCCCATTCTTCTT | 22 | 5 | 340 | 361 | + |
| TAS3_DV105041:1 | TCGCATCCTCATCTTAATCTC | 21 | 4 | 237 | 257 | + |
| TAS3_DV105041:1 | TTGACTTGTTGCCTTTCGTTC | 21 | 4 | 319 | 339 | + |
| TAS3_DV105041:1 | CTTGTTGCCTTTCGTTCCTCT | 21 | 4 | 323 | 343 | + |
| TAS3_DV105041:1 | ATGGGGTAGGTAGAGGAACGA | 21 | 4 | 334 | 354 | - |
| TAS3_DV105041:1 | TTAGGAAGACGAAGAGAAAGCC | 22 | 3 | 206 | 227 | - |
| TAS3_DV105041:1 | TTTTTTGACTTGTTGCCTTTC | 21 | 3 | 315 | 335 | + |
| TAS3_DV105041:1 | GTTCCTCTACCTACCCCATTC | 21 | 3 | 336 | 356 | + |
| TAS3_DV105041:1 | TCTTGACCTTGTAAGACCTTT | 21 | 3 | 358 | 378 | + |
| TAS3_DV105041:1 | TGAGGATGCGACACTCATCGT | 21 | 2 | 227 | 247 | - |
| TAS3_DV105041:1 | TCCTCATCTTAATCTCGGTGCT | 22 | 2 | 242 | 263 | + |
| TAS3_DV105041:1 | TTGCCTTTCGTTCCTCTACCT | 21 | 2 | 327 | 347 | + |
| TAS3_DV105041:1 | TTCTTGACCTTGTAAGACCT | 20 | 2 | 357 | 376 | + |
| TAS3_DV105041:1 | TTCTTGACCTTGTAAGACCTT** | 23 | 2 | 357 | 377 | + |
| TAS3_DV105041:1 | AGACCTTTTCTTGACCTTGTA | 21 | 2 | 371 | 391 | + |
| TAS3_DV105041:1 | TTCTTGACCTTGTAAGACCCCG | 22 | 2 | 378 | 399 | + |
| TAS3_DV105041:1 | TTGACCTTGTAAGACCCCGTG | 21 | 2 | 381 | 401 | + |
| TAS3_DV105041:1 | TTGACCTTGTAAGACCCCGTGT | 22 | 2 | 381 | 402 | + |
| TAS3_DV105041:1 | TAAGACCCCGTGTTATCTC | 19 | 2 | 390 | 408 | + |
| TAS3_DV105041:1 | AGACGTAAGAGATAACACGGGGTC | 24 | 2 | 393 | 416 | - |
| TAS3_DV105041:1 | AAGCTTTGTTTGGTCTCCTTC | 21 | 2 | 463 | 483 | + |
| TAS3_DV105041:1 | AGAAGGAGACCAAACAAAGCT | 21 | 2 | 464 | 484 | - |
| TAS3_DV105041:1 | TTTGTTTGGTCTCCTTCTTCT | 21 | 2 | 467 | 487 | + |
| TAS3_DV105041:1 | TTGTTTGGTCTCCTTCTTCTT | 21 | 2 | 468 | 488 | + |
| TAS3_DV105041:1 | TAAGAAGGACCACTAAACATG | 21 | 1 | 174 | 194 | - |
| TAS3_DV105041:1 | TTAGGAAGACGAAGAGAAAGCCAAAGCCATA | 31 | 1 | 197 | 227 | - |
| TAS3_DV105041:1 | TTTCTCTTCGTCTTCCTAACG | 21 | 1 | 209 | 229 | + |
| TAS3_DV105041:1 | TGCGACACTCATCGTTAGGAA | 21 | 1 | 221 | 241 | - |
| TAS3_DV105041:1 | ATCCTCATCTTAATCTCGGTG | 21 | 1 | 241 | 261 | + |
| TAS3_DV105041:1 | ACTTGTTGCCTTTCGTTCCTC | 21 | 1 | 322 | 342 | + |
| TAS3_DV105041:1 | TTGTTGCCTTTCGTTCCTCTA | 21 | 1 | 324 | 344 | + |
| TAS3_DV105041:1 | CTTTCGTTCCTCTACCTACCCC | 22 | 1 | 331 | 352 | + |
| TAS3_DV105041:1 | TTCGTTCCTCTACCTACCCCA | 21 | 1 | 333 | 353 | + |
| TAS3_DV105041:1 | TTCCTCTACCTACCCCATTC | 20 | 1 | 337 | 356 | + |
| TAS3_DV105041:1 | AAGAATGGGGTAGGTAGAGGAA | 22 | 1 | 337 | 358 | - |
| TAS3_DV105041:1 | TACCCCATTCTTCTTGACCTTG | 22 | 1 | 347 | 368 | + |
| TAS3_DV105041:1 | TTCTTGACCTTGTAAGACC | 19 | 1 | 357 | 375 | + |
| TAS3_DV105041:1 | TACAAGGTCAAGAAAAGGTCT | 21 | 1 | 371 | 391 | - |
| TAS3_DV105041:1 | TTCTTGACCTTGTAAGACC | 19 | 1 | 378 | 396 | + |
| TAS3_DV105041:1 | TGACCTTGTAAGACCCCGTGT | 21 | 1 | 382 | 402 | + |
| TAS3_DV105041:1 | TTGACCTTGTAAGACCCCGTGTTA | 24 | 1 | 381 | 404 | + |
| TAS3_DV105041:1 | TGTTATCTCTTACGTCTTTAT | 21 | 1 | 400 | 420 | + |
| TAS3_DV105041:1 | TGTTTGGTCTCCTTCTTCTTT | 21 | 1 | 469 | 489 | + |
| TAS3_DV105041:1 | TCCTCCTTCCTTGTCTATCCC | 21 | 1 | 505 | 525 | + |
| TAS3_DV105041:1 | TTCCTTGTCTATCCCTCCTGA | 21 | 1 | 511 | 531 | + |
| TAS3_DV105041:1 | ATCCCTCCTGAGCTGTTGATTT | 22 | 1 | 521 | 542 | + |
| TAS3_DV105041:1 | TCCCTCCTGAGCTGTTGATTT | 21 | 1 | 522 | 542 | + |

*, ** show D7 and D8 tasi*ARFs*, respectively.

**Table S8**. List of the primers used for RT-qPCR analysis.

| **Target candidate** | **Accession no.** | **Forward Primer (5'-3')** | **Reverse primer (5'-3')** |
| --- | --- | --- | --- |
| *ARF2* | Solyc03g118290.2.1 | AGTAGTTGTAGACACTTCAC | TTAACCCAGCTAGCAGGTGCT |
| *ARF3* | Solyc02g077560.2.1 | CAGACTTTGGGGAACCTTCT | AGCCTGGAACTTTGATGCTGG |
| *ARF4* | Solyc11g069190.1.1 | TGAGGAGTCAATAAGATCCTC | GGAAATTAGTACTGTAGGAGGG |
| *ARF8* | Solyc02g037530.2.1 | AATTGGGGCAGATGTTCGGT | CAATCAGTACTCCAGCGATCCA |
| *GAMYBL1* | Solyc01g009070.2.1 | CAACTCCTCTTCAGAGCCCA | ATGCTGTGTCTTGGGACTGG |
| *Cystatin* | Solyc00g071180.2.1 | GCAGTGATGGCAAACGTAGG | CACCTTCAGTGCCCTCAAAT |
| *PMEI* | Solyc03g112990.1.1 | GAGTTGCTGAATGTGGGTAC | AGGCCAAAAAGAAGTGCATGTG |
| *Cysteine protease* | Solyc07g053460.2.1 | TGCGGACATGACAAATCGAGA | ACAACCCCATCCTTCCTCCA |
| *Polygalacturonase* | Solyc07g044870.2.1 | CAGGTCAGCCAGGAGTGTTT | ACTCAAGTGGTGCCTTGCAT |
| *α-tubulin* | Solyc04g077020.2.1 | TGAGGTCTTCTCACGCATTGACCA | AATCCTTCTCGAGGGCAGCAAGAT |
| *CAC* | SGN-U314153 | CCTCCGTTGTGATGTAACTGG | ATTGGTGGAAAGTAACATCATCG |
| miR159 | MIMAT0000177 | TTTGGATTGAAGGGAGCTCTA | mRQ 3' primer* |
| miR390 | MIMAT0035479 | AAGCTCAGGAGGGATAGCG | mRQ 3' primer |
| miR319 | MIMAT0035479 | TTGGACTGAAGGGCGCTC | mRQ 3' primer |
| miR156 | MIMAT0009138 | TTGACAGAAGATAGAGAGCAC | mRQ 3' primer |
| miR167 | MIMAT0007917 | TGAAGCTGCCAGCATGATCTA | mRQ 3' primer |
| miR396 | MIMAT0035455 | TTCCACAGCTTTCTTGAACTG | mRQ 3' primer |
| miR#M | no entry | TCCGACTGAGAATTGCAAAGG | mRQ 3' primer |
| miR#A | no entry | ATGGGTAGCACAAGGATTAATG | mRQ 3' primer |
| miR#A* | no entry | TTCTCGTGAATCCTTCGGCTA | mRQ 3' primer |
| miR#B | no entry | TAACTTCGTCTAGCTCGCCTTC | mRQ 3' primer |
| miR#B* | no entry | AGGGGAGATAGATGAAGTTAGG | mRQ 3' primer |
| miR#C | no entry | TGTCGCAGATGACTTTCGC | mRQ 3' primer |
| miR#C* | no entry | ACGAGAGTCATCTGTGACAGG | mRQ 3' primer |
| miR#D | no entry | TGGGGAGGGTGGTGTGTAC | mRQ 3' primer |
| miR#D* | no entry | TGCGTACATCCTACCCTCC | mRQ 3' primer |
| D7 | no entry | TTCTTGACCTTGTAAGACCC | mRQ 3' primer |
| D8 | no entry | TTCTTGACCTTGTAAGACCTT | mRQ 3' primer |

* mRQ 3' primer was provided by the Mir-X™ miRNA First-Strand Synthesis and SYBR® qRT-PCR kit (Clontech)

**Table S9**. List of the primers used for 5ˊ-RACE analysis.

| **Target candidate** | **Accession no.** | **GeneRacer 5' primer (5'-3')** | **Gene-specific 3' primer (5'-3')** |
| --- | --- | --- | --- |
| *ARF2* | Solyc03g118290.2.1 | CGACTGGAGCACGAGGACACTGA | TTAACCCAGCTAGCAGGTGCT |
| *ARF3* | Solyc02g077560.2.1 | CGACTGGAGCACGAGGACACTGA | AGCCTGGAACTTTGATGCTGG |
| *ARF4* | Solyc11g069190.1.1 | CGACTGGAGCACGAGGACACTGA | GGAAATTAGTACTGTAGGAGGG |
| *ARF8* | Solyc02g037530.2.1 | CGACTGGAGCACGAGGACACTGA | CAATCAGTACTCCAGCGATCCA |
| *GAMYBL1* | Solyc01g009070.2.1 | CGACTGGAGCACGAGGACACTGA | ATGCTGTGTCTTGGGACTGG |
| *Cystatin* | Solyc00g071180.2.1 | CGACTGGAGCACGAGGACACTGA | CACCTTCAGTGCCCTCAAAT |
| *PMEI* | Solyc03g112990.1.1 | CGACTGGAGCACGAGGACACTGA | AGGCCAAAAAGAAGTGCATGTG |

**Table S10**. List of the DIG-labeled oligo-probes used for in situ hybridization.

| **Target candidate** | **Probe sequence (5'-3')** |
| --- | --- |
| miR159 | TAGAGCTCCCTTCAATCCAAA |
| *GAMYBL1* | ACTGATGCAGAGAATTGACTT |
| *PMEI* | AGGCCAAAAAGAAGTGCATGTG |
| *Cystatin* | CACCTTCAGTGCCCTCAAAT |
| miR122a | TGGAGTGTGACAATGGTGTTTG |
