# Supplementary material for: Identification of miRNAs with potential roles in regulation of anther development and male-sterility in 7B-1 male-sterile tomato mutant
Source: BMC Genomics. 2015 Oct 28;16:878. doi: 10.1186/s12864-015-2077-0 (PMC4625851; doi:10.1186/s12864-015-2077-0)
Supplement: Additional file 2: Figure S1. — Hairpin structures of new miRNA precursors. Figure S2. 5′- RACE validation of tasiARFs target genes in 7B-1 anther and stem. Figure S3. RT-qPCR validation of tasiARFs target genes in 7B-1 anther and stem. Figure S4. RT-qPCR validation of cysteine protease and polygalacturonase in 7B-1 anthers and stem. Figure S5. RT-qPCR validation of miR159 and GAMYBL1 in GA-treated 7B-1 anthers. Figure S6. Cytological study of anther development in 7B-1 and WT. (DOCX 4243 kb) [file 12864_2015_2077_MOESM2_ESM.docx]

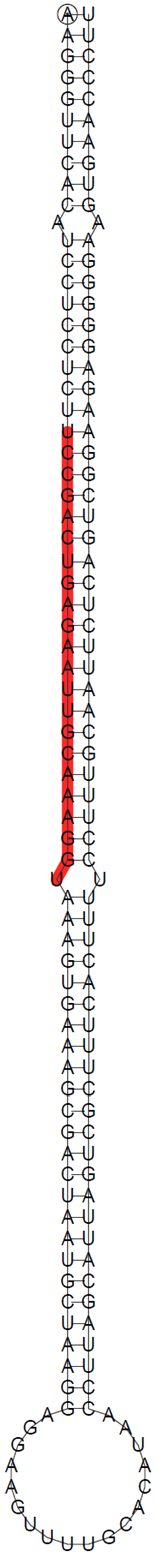


miR#W


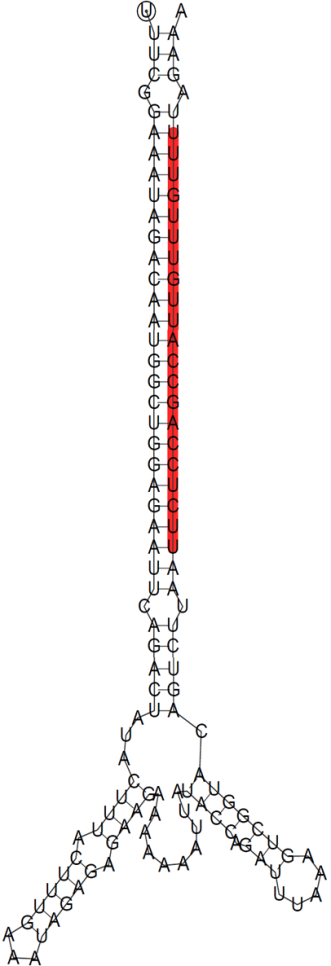


miR#M


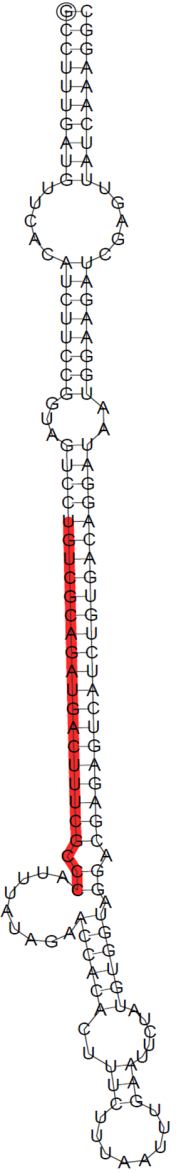


miR#A


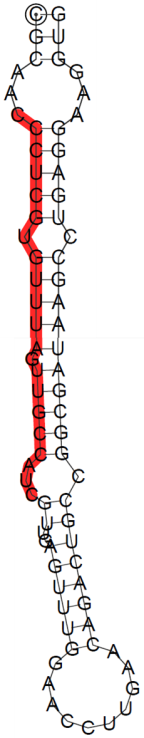


miR#B

**Figure S1** Hairpin structures of new miRNA precursors as determined by the RNAfold program. The miRNA sequences are in highlighted in red.


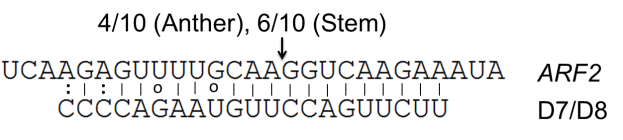

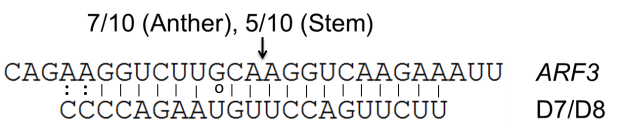


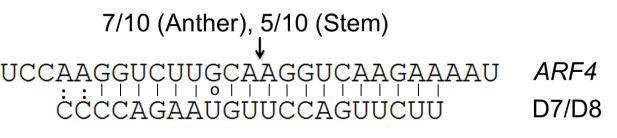


**Figure S2** 5ˊ- RACE validation of tasi*ARFs* target genes in *7B-1* anther and stem. Gene transcripts are in 5ˊ-3ˊ and tasi*ARFs* in 3ˊ-5ˊdirections. The arrows indicate the cleavage sites of target mRNA and numbers above them indicate frequency (out of 10) of sequences found at the exact miRNAs cleavage sites. Watson-Crick pairing (vertical dashes), G-U wobble pairing (circles), and other mismatches (:) are indicated.

**Figure S3** RT-qPCR validation of tasi*ARFs* target genes in *7B-1* anther and stem. Expression changes are presented as normalized fold changes between *7B-1* and WT reference tissue. Positive and negative values indicate up- and down-regulation of the expression, respectively. Two-fold threshold was considered as a cutoff value for significant changes in the expression. Error bars represent standard errors of three biological replicates based on DMNRT (p = 0.05).

**Figure S4** RT-qPCR validation of *cysteine protease* and *polygalacturonase* in *7B-1* anthers and stem. Expression changes are presented as normalized fold changes between *7B-1* and WT reference tissue. Positive and negative values indicate up- and down-regulation of the gene expression, respectively. Two-fold threshold was considered as a cutoff value for significant changes in the expression. Error bars represent standard errors of three biological replicates based on DMNRT (p = 0.05).

**Figure S5** RT-qPCR validation of miR159 and *GAMYBL1* in GA-treated *7B-1* anthers. Expression changes are presented as normalized fold changes between GA-treated and H_2_O-treated anthers (reference tissue). Positive and negative values indicate up- and down-regulation of the expression, respectively. Two-fold threshold was considered as a cutoff value for significant changes in the expression. Error bars represent standard errors of three biological replicates based on DMNRT (p = 0.05).


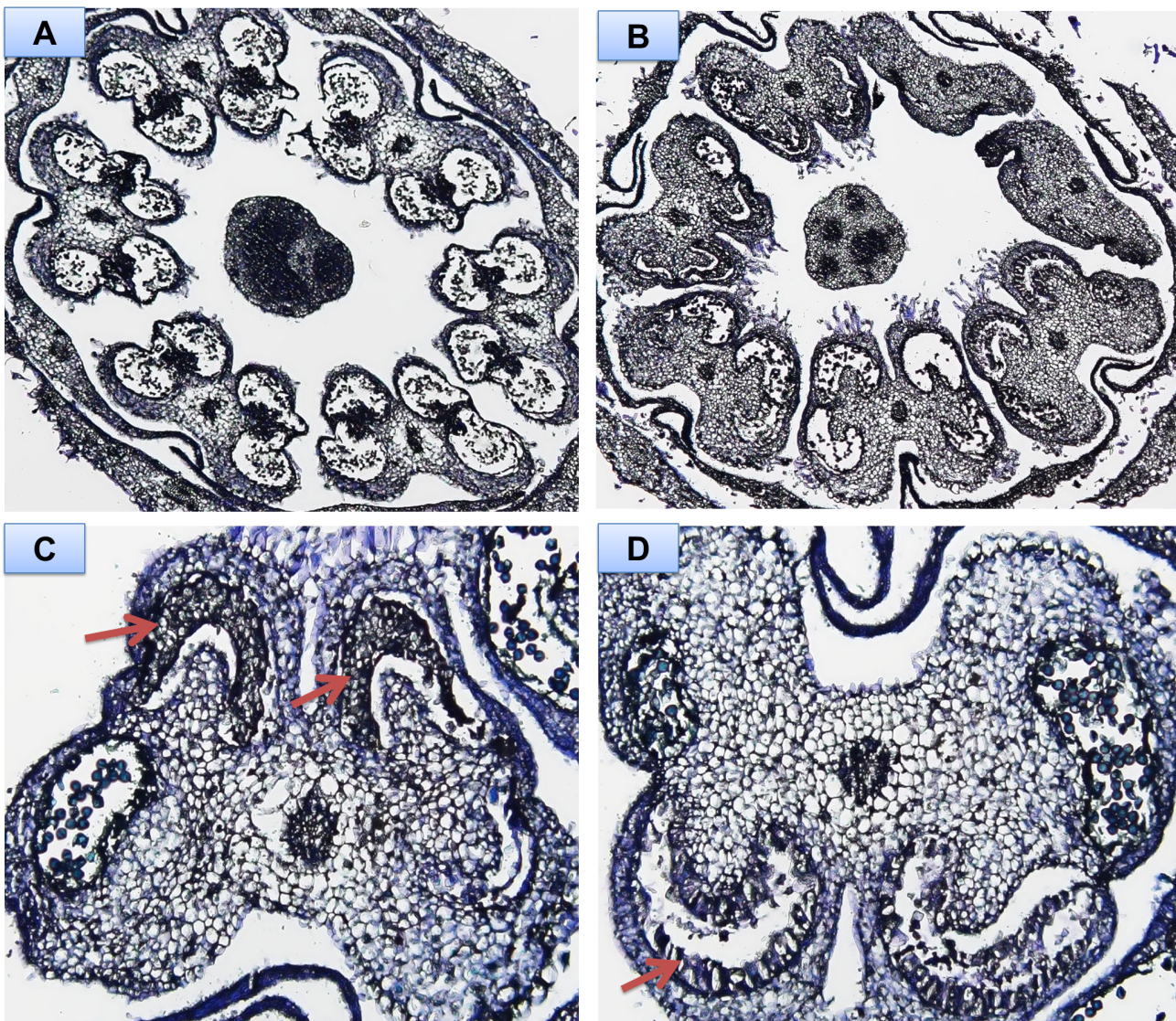


**Figure S6** Cytological study of anther development in *7B-1* and WT. A: micrograph of WT anthers, where anther development is synchronized within a flower bud. B: micrograph of *7B-1* anthers, where anther developments is not synchronized within anther lobes and different anthers. C: micrograph of *7B-1* anther showing arrested (indicated by arrows) and free uninucleate microspores. D: micrograph of *7B-1* anther showing vacuolated (indicated by arrow) and degenerated tapetum.
